# Chronic high consumption of energy drinks and cardiovascular risk in adolescents—results of the EDKAR-study

**DOI:** 10.1007/s10654-025-01292-z

**Published:** 2025-08-23

**Authors:** Juliane Menzel, Fabian Spinka, Maria J. Pie, Andrea Deichl, Sven Knüppel, Anke Ehlers, Britta Nagl, Frank Edelmann, Cornelia Weikert

**Affiliations:** 1 Department Food and Feed Safety in the Food Chain, German Federal Institute for Risk Assessment, Max-Dohrn-Straße 8-10, 10589 Berlin, Germany; 2 Institute of Social Medicine, Epidemiology and Health Economics, Charité – Universitätsmedizin Berlin, Freie Universität Berlin and Humboldt-Universität zu Berlin, Berlin, Germany; 3 German Center for Cardiovascular Research (DZHK), Partner Site Berlin, Berlin, Germany; 4 Department of Cardiology, Angiology and Intensive Care Medicine, Deutsches Herzzentrum der Charité (DHZC) – Medical Heart Center of Charité and German Heart Institute Berlin, Berlin, Germany; 5 Charité – Universitätsmedizin Berlin, Freie Universität Berlin and Humboldt-Universität zu Berlin, Berlin, Germany

**Keywords:** Energy drink, Adolescents, Cardiovascular system, Heart rate, Blood pressure, Electrocardiographic, Echocardiographic

## Abstract

In recent years, acute cardiovascular effects of high energy drink (ED) consumption have been described, but no data are available on chronic high consumption of EDs and cardiovascular risk in adolescents. As a first study, the present study investigated differences in a variety of cardiological parameters in adolescents (aged 15–18 years) with a chronic high consumption of EDs (ED consumption: ≥ four days/week for ≥ last 12 months, > 3 mg caffeine from EDs/kg bodyweight/day) compared to a control group. In study phase 1 of the cross-sectional EDKAR-study, data from 5100 pupils in Berlin (Germany) on their ED consumption and lifestyle factors were assessed using an online questionnaire. Based on these, adolescents with a chronic high ED consumption (*n* = 97) and a control group (*n* = 160) were cardiologically examined at Charité - Universitätsmedizin Berlin. Blood pressure, heart rate, electrocardiographic and echocardiographic parameters were assessed. Cardiological risk factors like educational background, smoking, alcohol consumption and sleep duration were investigated. The study noticed no significant and/or clinically relevant differences in any of the cardiological parameters e.g., heart rate (Chronic high ED consumption: Geometric mean (95%-CI): 74.8 BPM (68.5–81.8) vs. control group: 71.9 BPM (65.2–79.2), *p* = 0.23). However, half of the high consumers reported having experienced adverse effects after consuming EDs. Furthermore, adolescents with chronic high ED consumption reported a considerably higher intake of alcohol, higher smoking rates and shorter sleep duration in comparison to the control group. Accordingly, chronic high ED consumption is associated with lifestyle factors with a potential negative impact on the cardiovascular system.

**Supplementary Information:**

The online version contains supplementary material available at 10.1007/s10654-025-01292-z.

## Introduction

Energy drinks (EDs) are highly caffeinated beverages that contain caffeine along with other ingredients such as taurine, glucuronolactone, and inositol [1]. Since their introduction to the market in 1987, EDs have become increasingly popular across a wide range of consumers [2]. Particularly among adolescents EDs have gained widespread popularity, suggested by a survey of the European Food Safety Authority (EFSA) [3]. In addition, a recently published survey from 2024 (*n* = 1000, aged 14–18 years) conducted by the Forsa research institute on behalf of the consumer organization foodwatch, shows that almost one in three young people in Germany regularly consume EDs [4]. According to the survey, 19% of responders consume EDs several times a month, 8% several times a week and 2% daily [4].

Focusing upon EDs, in recent years potential health effects of EDs has been proposed. Beside to the described association between ED consumption and mental health risk (depressive symptoms) or adverse metabolic effects (obesity or type 2 diabetes) [5], multiple studies conducted in adults suggested associations between ED consumption and cardiovascular health. Indeed, recent reviews [6–10] and studies [11–16] indicated that high acute ED consumption might affect the cardiovascular system, influencing cardiovascular parameters such as heart rate, systolic blood pressure (SBP), diastolic blood pressure (DBP), and electrocardiographic measures like the QTc interval. Further, a recent review of case reports by Costantino et al. showed a high prevalence of adverse cardiovascular effects after ED consumption in adults [17]. In addition, a review of case reports has highlighted a possible association between acute (excessive) ED consumption (various quantity of ED consumption from one ED can to several liters), often in combination with alcohol and drug use, and an increased risk of cardiovascular complications, including abnormal heart rhythms such as atrial and ventricular fibrillation, myocardial ischemia, tachycardia, ST-segment elevation and QT prolongation [6, 18–21].

Despite the fact that minors are considered one of the main consumer groups of EDs, there is only limited research available as to the potential cardiovascular health effects of high ED- or caffeine consumption in this group. In fact, EFSA reported in 2015 that the data available for children and adolescents regarding the relationship between caffeine intake and health effects are insufficient to derive a safe intake level [22]. However, EFSA concludes that the single-dose acute caffeine intake, which is of no health concern for adults, can also be considered harmless for children and adolescents due to their higher metabolization rate [22]. Therefore, EFSA suggested that caffeine intake levels of 3 mg/kg body weight (BW)/day provide a basis for calculating caffeine intake levels of no concern for children and adolescents [22].

So far, there have only been limited studies from independent study populations available investigating the acute moderate use of EDs (up to 3 mg caffeine from EDs/kg BW/day [23–27] or 250-ml ED can [28]) with regard to cardiovascular health in children and adolescents. In fact, various publications from the EDUCATE-study (Energy-Drinks – Unexplored Cardiovascular Alterations in TEens and TwEens) have been published in recent years [23–27]. In line with the results from adult populations acute moderate consumption of EDs increases the SBP and/or DBP in adolescents [23, 24, 28], affects heart rhythm [26] and changes parameters for measuring arterial stiffness [27]. Further, a recently published review of case reports on minors (aged 8 to 17 years) suggested that acute and partly excessive ED consumption may be associated with adverse health effects, particularly affecting the cardiovascular and neuropsychiatric systems e.g. chronic headache or seizures [29]. So far chronic high consumption of EDs has not been investigated with regard to cardiovascular health in adolescents. Therefore, the present study aimed to investigate blood pressure, heart rate and a wide range of electrocardiographic and echocardiographic parameters in adolescents with chronic high ED consumption compared to a control group.

## Methods

The present cross-sectional study on chronic high consumption of EDs and cardiovascular risk (EDKAR-study) examines blood pressure, heart rate and a wide range of electrocardiographic and echocardiographic parameters in adolescents aged between 15 and 18 years. The EDKAR-study was conducted in two phases. In the first study phase, a comprehensive online questionnaire was used to collect data on the consumption of EDs in order to identify adolescents with chronically high ED consumption and a control group for the second study phase (cardiological examination). Participants gave their written informed consent. Study procedures were approved by the Ethics Committee of Charité University Medical Center Berlin (no. EA2/176/21) and were conducted in accordance with the Declaration of Helsinki. The study was registered in the German Clinical Trials Register (DRKS) DRKS00026698 (Registered 15 November 2021). The adolescents received a monetary incentive (50€) for the cardiological examination in study phase 2.

### Design of the EDKAR-study

**Fig. 1:**
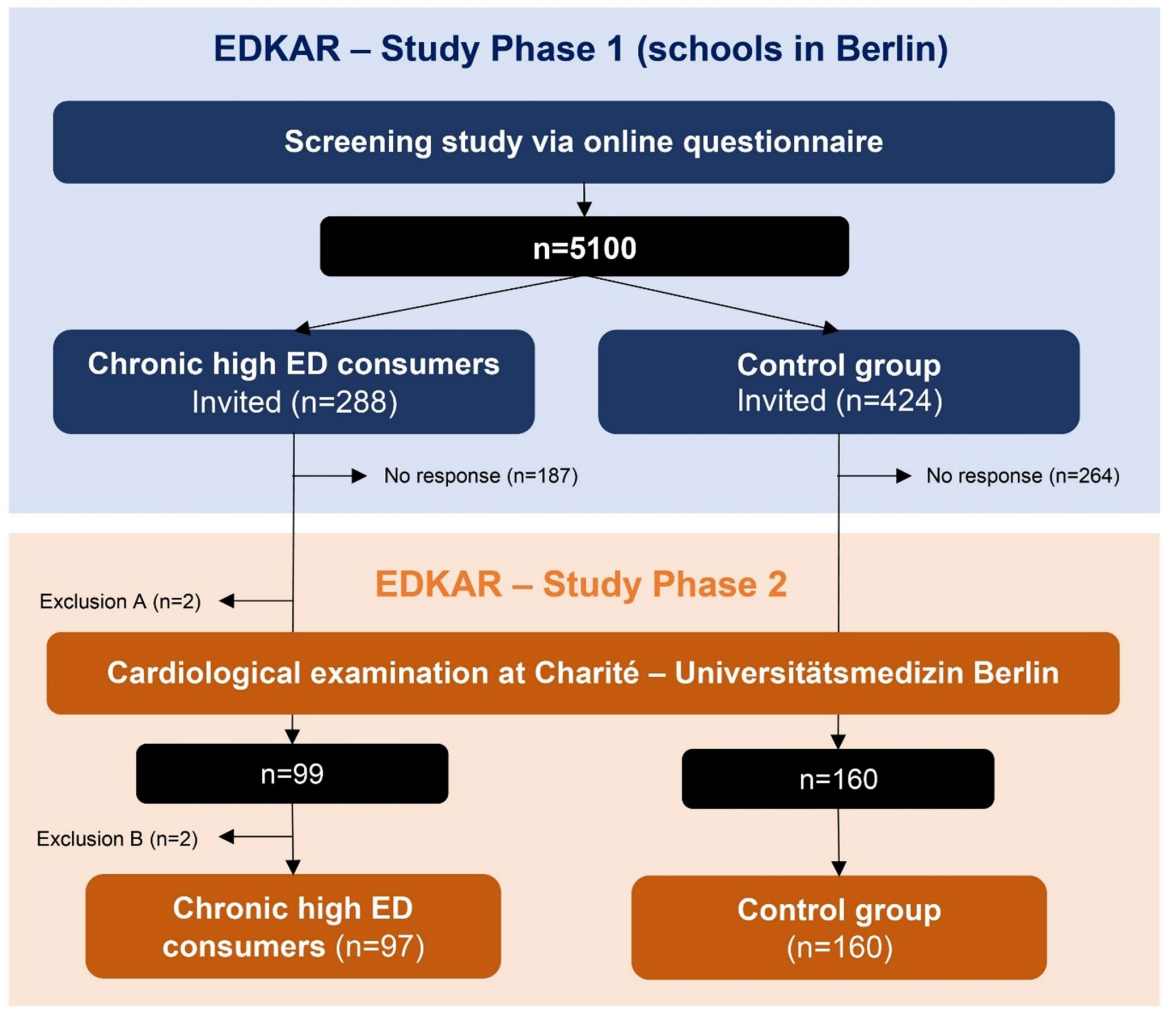
Flowchart of the study on chronic high consumption of EDs and cardiovascular risk (EDKAR-study)

The EDKAR-study comprised two study phases. The first study phase used a comprehensive online questionnaire to assess data about the consumption of EDs, as well as leisure and health behaviour (aim: *n* = 5000 pupils) with the aim to identify adolescents with chronic high ED consumption as well as a control group. The survey of pupils took place in Berlin schools after approval was granted by the Senate Department for Education, Youth and Family. In detail, during the recruitment in study phase 1 over the entire project period, a total of 156 randomly selected school principals across Berlin were contacted by telephone and/or email and asked to enable participation of suitable classes (aged 15–18 years) in the study phase 1. Once the school principals had agreed, the study team presented the EDKAR-study to the pupils in person. After giving their written consent, the adolescents received personalized access to the online questionnaire. During the study phase 1, potential participants (chronic high ED consumers or control group) were continuously identified by their answers in the online questionnaire on the consumption of EDs or other beverages containing caffeine. Accordingly, in the present study possible chronic high ED consumers were defined as adolescents who had consumed EDs (1) on at least four days per week [3], (2) for at least the last 12 months and (3) had exceeded a daily caffeine intake from EDs of more than 3 mg caffeine from EDs/ kg BW/ day [22]. In contrast, the control group were defined as adolescents, (1) who had not consumed any EDs in the last 12 months and (2) had not consumed more than 80 mg of caffeine per week from other caffeinated drinks, i.e. coffee drinks, black/green tea, drinking chocolate, cola/ mate, iced tea or pre-workout boosters. Accordingly, identified chronic high ED consumers and controls were given an invitation by letter for study phase 2 by the teaching staff. The pupils responded to this invitation individually by phone, email or enclosed reply letter. The study team contacted interested study participants by telephone to ensure that the participants were fully informed about study phase 2 (cardiological examination). After the telephone call, the (parental) study information and (parental) consent forms were sent by post or email. Then, a possible appointment for the cardiological examination in the study centre at Charité - Universitätsmedizin Berlin was scheduled. Initially a matching factor of 1:2 by age and sex was aimed for participants with chronic high ED consumption compared to the control group, but due to the individual self-determined feedback of the pupils, the planned age- and sex-matched enrolment in study phase 2 had to be waived during the study. If implausible information on the consumption of EDs was found during the identification process, consumption of EDs were assessed again during the telephone call. As a result, two participants had to be excluded from study phase 2 (Fig. 1) as the adolescents no longer met the inclusion criterion for chronic high ED consumption of > 3 mg caffein from EDs/ kg BW /day (Exclusion A). In study phase 2 blood pressure, heart rate and a wide range of electrocardiographic and echocardiographic parameters of chronic high ED consumers (aim: *n* = 50–100) in comparison to a control group (aim: *n* = 100–200) were assessed at Charité - Universitätsmedizin Berlin.

### Identification of potential chronic high ED consumers and control group

For the identification of chronic high ED consumption, the data from the online questionnaire was used to check which participants had consumed EDs at least four days a week during the last 12 months. In addition, the total quantity of EDs (in mL) and the corresponding consumption of caffeine in relation to BW were used to identify participants who consumed > 3 mg caffeine from EDs/ kg BW/ day. BW was determined as self-reported in a free field. Alternatively, BW categories (40–44 kg, 45–49 kg, 50–53 kg, 54–59 kg, 60–64 kg, 65–69 kg, 70–74 kg, 75–80 kg, 81–89 kg, 90–106 kg, 107–133 kg, > 134 kg) were also offered and the lowest limit of the categories was used to calculate the caffeine intake from EDs per kilogram of BW. To identify a possible control group, data of the online questionnaire was used to check which participants had not consumed any EDs in the past 12 months. To qualify for the control group, the intake of caffeine from other caffeinated beverages should not exceed 80 mg caffeine per week. For the calculation of the weekly caffeine intake, the frequency/consumption of coffee drinks (90 mg caffeine/cup), black/green tea (38 mg caffeine /cup), drinking chocolate (30 mg caffeine /cup), cola/mate soft drinks (31 mg caffeine /glass), iced tea (10 mg caffeine /glass) or pre-workout boosters/training boosters (225 mg caffeine /serving) were used.

### Final study population of the EDKAR-study

The EDKAR-study was conducted from March 2022 until May 2024. In total, over the entire project period 359 classes from 29 schools supported the EDKAR-study and were included in study. As depicted in Fig. 1, the first study phase (conducted from March 2022 until January 2024) was completed with a total of 5100 participants (with complete data sets). Among the 5100 participants, 288 chronic high consumers of EDs and 424 controls were identified and invited to study phase 2 for a cardiological examination. In study phase 2, blood pressure, heart rate and a wide range of electrocardiographic and echocardiographic parameters of 99 chronic high ED consumers and 160 controls were assessed at Charité - Universitätsmedizin Berlin from August 2022 until May 2024. The median time from online questionnaire fill-in during study phase 1 to cardiological examination was 66 days (IQR: 50–98).

The final study population in the present study comprised 97 chronic high EDs consumers, as two participants had to be excluded retrospectively due to missing inclusion criteria (ED caffeine < 3 mg caffeine/ kg BW/ day) and pre-existing cardiological disease (Exclusion B).

Study phase 1 of the EDKAR-study was completed by 5100 participants (with complete data sets). Among the 5100 participants, 288 chronic high ED consumers and 424 associated controls were identified in the data set and invited to study phase 2. In study phase 2, 99 chronic high ED consumers and 160 controls were cardiologically assessed at Charité - Universitätsmedizin Berlin. The final study population in the present study comprised 97 chronic high ED consumers and 160 controls.

No response: Participants were invited to study phase 2, but did not respond not to the invitation and were not cardiologically examined. Exclusion A: After telephone verification of ED consumption, participants (*n* = 2) did not meet the inclusion criterion (> 3 mg caffein from EDs/kg BW/day) and were excluded (not cardiologically examined). Exclusion B: Participants had to be excluded retrospectively (cardiologically examined) due to missing inclusion criteria (*n* = 1) and pre-existing cardiological disease (*n* = 1).

### Cardiological examination

The cardiological examination in study phase 2 was carried out at the Charité - Universitätsmedizin Berlin. Clinical investigators have been blinded with regard to group affiliation (chronic high consumers of EDs vs. controls). Blood pressure (SBP and DBP) was measured by automated blood pressure monitors (BOSO carat professional) on the left upper arm, sitting, at heart level after 5 min of rest and 2 min later. The mean of SBP and DBP of both measurement time points were used in the present study. In order to further investigate blood pressure, all participants were classified according to the German Hypertension League into the groups of optimal blood pressure (SBP < 120 mmHg and DBP BP < 80 mmHg), normal blood pressure (SBP 120–129 mmHg and/or DBP 80–84 mmHg), high normal blood pressure (SBP 130–139 mmHg and/or DBP 85–89 mmHg) or high blood pressure (SBP ≥ 114 mmHg and/or DBP ≥ 90 mmHg) [30].

Electrocardiograms (ECGs) obtained in a resting state with the patient in a supine position. Standard 12-lead ECGs were performed using equipment e.g., a 12-lead digital electrocardiograph (Schiller CARDIOVIT AT-102 plus), with leads placed according to the standard. All ECGs were archived in pCRF for subsequent analysis. The analysis of the ECG included heart rate, rhythm, PQ interval, QT interval, QRS duration, the presence of AV block, the presence of any bundle branch block, the observation of discordant negative T waves, and the detection of ST segment abnormalities. Bazett’s formula was applied for QTc interval correction [31]. In addition, pathological Q waves were reported if observed.

Transthoracic echocardiography (TTE) was performed using a GE Vivid E95 or a Philips Epiq 7G ultrasound system and was analysed with Tomtec Image-Com (Version 2.51.4). Imaging was carried out by experienced echocardiographers trained in cardiac sonography. The patients were positioned in the left lateral position, and images were obtained using standard views, including parasternal, apical, and subcostal windows. The examination was conducted in accordance with the recommendations of the European Association of Cardiovascular Imaging (EACVI) guidelines [32]. Doppler studies (pulsed-wave, continuous-wave, colour and tissue Doppler) were performed, as well as two-dimensional (2D) echocardiographic images were obtained to evaluate specific parameters, i.e. left ventricular ejection fraction (LVEF), left ventricular endiastolic diameter, (LVEDd), interventricular septum thickness (IVSd), Posterior wall thickness (PWED), left ventricular mass index (LVMI), left atrial volume four-chambers (LAVES (4CH)), left atrial volume two-chambers (LAVES (2CH)), left atrial volume mean from LAVES (4CH) and LAVES (2CH) (LAVES mean), left atrial volume index (LAVI), tricuspid annular plane systolic excursion (TAPSE), mitral annular plane systolic excursion (MAPSE), E-wave, A-wave, E/A, e’ (lateral, septal, mean) E/e’ (lateral, septal, mean), Global longitudinal strain were assessed. Echocardiographic recordings were digitally stored for further analysis. All participants were classified according to medical norm values/ reference values for the parameters PQ interval, QT interval, QRS duration, LVEF, LVEDd, IVSd, PWED, LVMI, LAVI, TAPSE, MAPSE, E/A, e‘ lateral, e‘ septal, e‘ mean, E/e’ mean, Global longitudinal strain in order to investigate differences in the classification between the chronic high ED users and the control group, accordingly for each parameter [33].

Furthermore, in study phase 2, anthropometric measurements (weight, height, and waist circumference) were collected by trained and quality-monitored personnel, with participants wearing only light underwear and no shoes. Body weight was assessed by an electronic digital scale (Soehnle professional) and the height was measured using a flexible anthropometer (Seca). The formula for body mass index (BMI) comprises weight in kilograms divided by height in meters squared. Waist circumference was defined as in the horizontal plane midway between the lowest ribs and the iliac crest.

### Power calculation

Since no studies on high ED consumers compared to a control group of adolescents are available to date, the power calculation was carried out using echocardiographic parameters from the study by Menci et al. (based on a study population under 25-year-olds) [34]. The power calculation of the EDKAR-study was conducted for different echocardiographic parameters. Accordingly, a difference of 5% or 10% between chronic high ED consumers in comparison to the control groups was considered clinically relevant, along with a level of significance of 5% and a power of 80% (Ratio 1:2). Based on the three echocardiographic parameters (difference 5%) of left ventricular ejection fraction (LVEF), tricuspid annular plane systolic excursion (TAPSE), mitral annular plane systolic excursion (MAPSE), a number of *n* = 18–65 chronic high ED consumers (depending on the parameter) and a number of *n* = 36–130 for the control group were calculated. If a difference of 10% was classified as clinically relevant, correspondingly fewer study participants were required for these parameters (*n* = 5–17 chronic high ED consumers; *n* = 11–34 control group). For the echocardiographic parameter global longitudinal strain (difference of 5%) 100 chronic high ED consumers and 200 adolescents in the control group were required. The echocardiographic parameter left ventricular mass index (LVMI) (a difference of 10%) required 85 chronic high ED consumers and 170 controls. The power calculations were performed using G*Power (G*power, version 3.1., Heinrich Heine University, Dusseldorf, Germany) without considering multiple testing for mean comparisons of two independent samples [35]. Taken together, according to the power calculation for various echocardiographic parameters, the EDKAR-study required 50–100 chronic high ED consumers and 100–200 controls for the cardiological examination in study phase 2. Thus, assuming that approximately 2–4% of the adolescents participating in the study phase 1 fulfil the criteria of chronic high ED consumers, in addition to an expected willingness to participate of 50% in the cardiological examination, at least *n* = 100–200 chronic high ED consumers and *n* = 200–400 controls needed to be identified and invited in study phase 2. Based on these assumptions, the EDKAR-study aimed to include at least *n* = 5000 pupils in study phase 1.

### Statistics

The present study (study phase 2 of the EDKAR-study) characterized the sample of adolescents with a chronic high ED consumption (*n* = 97) in comparison to a control group (*n* = 160), who have undergone cardiological examinations at Charité - Universitätsmedizin Berlin. Group differences were examined with regard to baseline characteristics e.g., physical activity, BMI and other lifestyle factors such as smoking and alcohol consumption. Due to the skewed distribution of all continuous variables, Mann-Whitney U-tests were used to compare the continuous variables between both groups, and a chi-square test was used for categorical variables. To investigate associations of a wide range of cardiological parameters between adolescents with a chronic high ED consumption compared to the control group, an analysis of variance (ANOVA) was performed for model 1 (unadjusted). Directed acyclic graphs (DAG) were used to estimate the minimal sufficient adjustment sets of potential confounders for the total effect of ED consumption on cardiological parameter (Supplemental Fig. 1), using browser-based environment DAGitty available online (https://www.dagitty.net) [36]. The multivariable adjusted analysis of covariance (ANCOVA) was conducted to detected differences between adolescents with a chronic high consumption of EDs in comparison to a control group in model 2 (adjusted for age, sex, BMI, physical activity, smoking status, smoking weed and alcohol consumption) and model 3 (additionally for school type). For the parameters LVMI and LAVI the body surface area accounted for the derivation (calculated from height and weight), therefore the adjustment of BMI of these parameters was waived in Model 2 and 3. All cardiological parameters were skewed, thus variables were log-transformed for ANOVA or ANCOVA, subsequently back-transformed and expressed as geometric means and 95%-confidence intervals (95%-CI). In addition, linear mixed models (adjusted for age, sex, BMI (except LVMI and LAVI), physical activity, smoking status, smoking weed and alcohol consumption) were used to investigate clustered effects of school type by modelling random effects that capture the variability between clusters.

For a more detailed investigation differences in blood pressure, heart rate, electrocardiographic and echocardiographic parameters several sensitivity analyses were conducted. First a median split (median: 4.5 mg caffeine from EDs/ kg BW/ day) procedure was carried out for caffeine intake based on EDs compared to the control group. In further sensitivity analyses, differences in blood pressure, heart rate, electrocardiographic and echocardiographic parameters between chronic high ED consumers and control groups were investigated in: only the upper 10% of the amount of caffeine intake from EDs of chronic high ED consumers, participants with a consumption ≥ 6 mg caffeine from EDs/ kg BW/ day and participants who had consumed EDs for more than two years. In addition, a sensitivity analysis was also performed using a median split of total caffeine intake from EDs and other caffeinated beverages (median: 6.65 mg caffeine/ kg BW/ day). According to these sensitivity analyses, Mann-Whitney U-tests were performed to investigate group differences. To investigate potential selection bias, baseline characteristics of all invited chronic high ED consumers (without response) were compared to the cardiologically examined chronic high ED consumers. The same procedure was performed with the control group, the invited control group (without response) were compared with the cardiologically assessed controls (see Fig. 1).

The statistical analyses were performed using SAS software, version 9.4 (SAS institute, Cary, NC, USA). A p-value < 0.05 was considered statistically significant. However, due to the multiple comparisons of cardiological parameters between adolescents with a chronic high ED consumption in comparison to a control group, the Bonferroni correction was applied by calculation of the original p-value (0.05) divided by the number of tests performed (216 tests). Thus, a *p* < 0.0002 was considered statistically significant.

## Results

### Study population and baseline characteristics

**Fig. 2:**
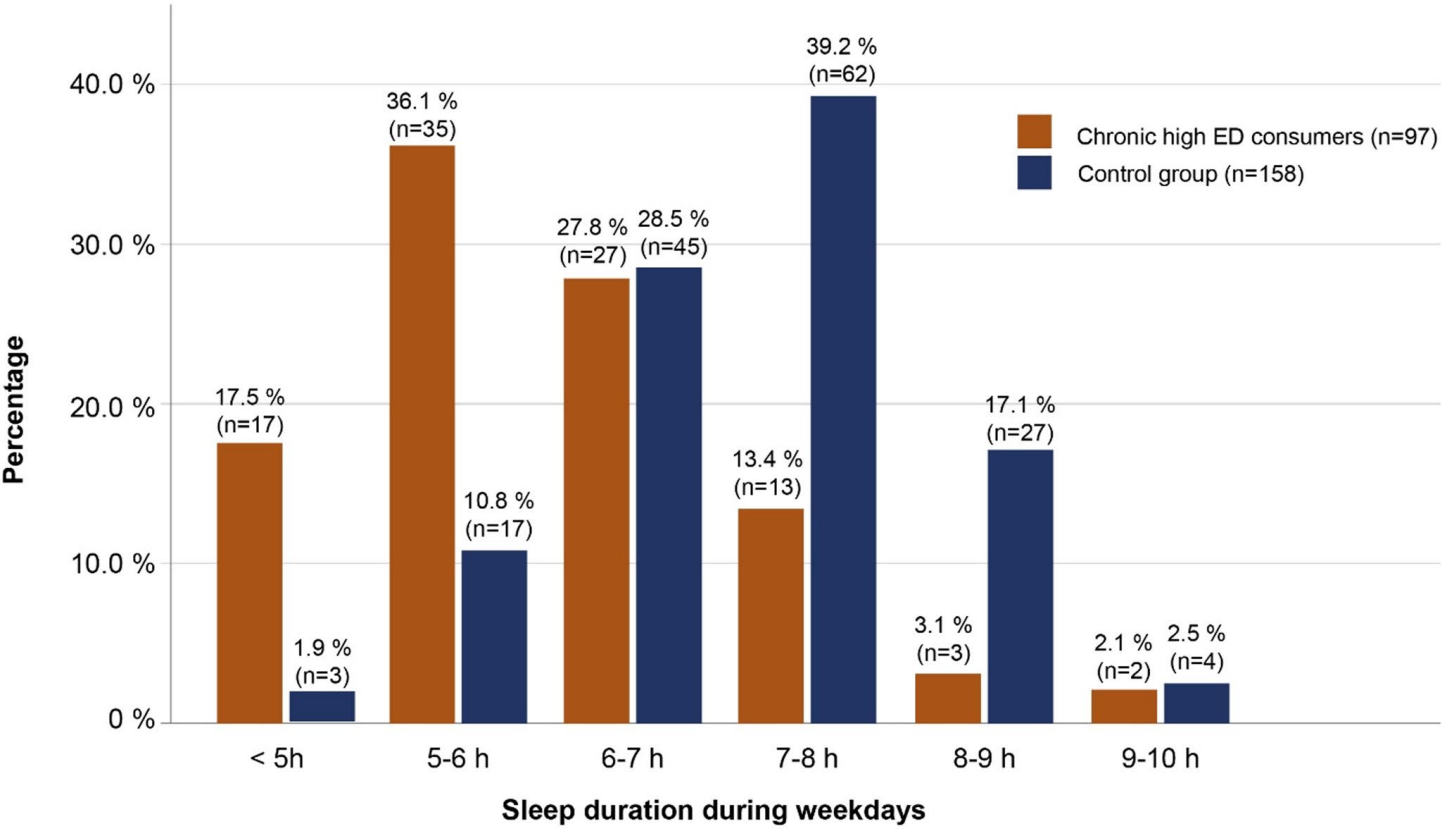
Sleep duration on weekdays between those with chronic high ED consumption (*n* = 97) and the control group (*n* = 158)

Table 1 shows the basic characteristics of cardiologically examined participants with chronic high ED consumption (*n* = 97) in comparison to the control group (*n* = 160) of study phase 2 (cardiological examination). We observed no differences in sex (Table 1). However, compared to the control group, participants with chronic high ED consumption had a higher BMI (*p* = 0.0006) and were more likely to be attending a vocational high school center. They also reported considerably more often to smoke, to smoke weed or consume alcohol (all *p* < 0.0001). In addition, 25.8% (*n* = 25) of chronic high ED consumers reported ‘sometimes’ mixing EDs with alcohol, while 14.4% (*n* = 14) did so ‘often’ or ‘always’ and 17.5% (n = 17) did so only ‘rarely’. With regard to the duration of sleep on weekdays the data showed that the chronic high ED consumers sleep for shorter durations (less than 6 hours: 53.6%, n = 52) than individuals in the control group (12.7%, n = 20), with the latter sleeping mainly 7-8 h (39.2%, n = 62) (Fig. 2). A similar picture emerges for the duration of sleep on the weekends, although around 20% in both groups reported sleeping longer compared to weekdays (10 h or more) (Supplemental Table 1). Even when smoking status and sleep duration were adjusted for other important influencing factors such as sex, age and school type, the influence of ED consumption on these factors remained.

**Table 1:** Basic characteristics of the participants in the study phase 2 of the EDKAR-Study

	*n*	Chronic high ED consumers (*n* = 97)	Control group (*n* = 160)	*p*-Values
Sex	257			*0.66*
Female		51.5% (50)	48.8% (78)	
Male		48.5% (47)	51.2% (82)	
Age [years]	257	16.0 (16.0–17.0)	16.0 (15.0-16.5)	*0.04*
BMI [kg/m^2^]	257	22.2 (19.7–24.6)	20.8 (19.0-22.9)	*0.0006*
Waist circumference	253			
Female [cm]		68.0 (65.0–74.0)	67.0 (63.0–71.0)	*0.10*
Male [cm]		76.0 (71.0–84.0)	74.0 (71.0–81.0)	*0.55*
Type of school	257			*< 0.0001*
Grammar school		14.4% (14)	33.8% (54)	
Secondary school		55.7% (54)	55.6% (89)	
Vocational high school center		29.9% (29)	10.6% (17)	
Born in Germany	256	82.3% (79)	91.9% (147)	*0.02*
Physical activity	253			*0.42*
Never		13.7% (13)	8.9% (14)	
< 1x per week		14.7% (14)	12.0% (19)	
1x per week		12.6% (12)	20.9% (33)	
2-6x per week		48.4% (46)	47.5% (75)	
Daily		10.5% (10)	10.8% (17)	
Smoking	250			*< 0.0001*
Smoking		50.5% (46)	1.9% (3)	
Ex-Smoking		14.3% (13)	3.1% (5)	
Non-Smoking		35.2% (32)	95.0% (151)	
Ever smoked Weed [yes]	255	44.2% (42)	5.0% (8)	*< 0.0001*
Alcohol consumption				
Ever consumed alcohol [yes]	254	75.0% (72)	46.2% (73)	*< 0.0001*
Alcohol consumption last 12 month	135			*< 0.0001*
1x per month or less		25.4% (18)	76.6% (49)	
2–3x per month		29.6% (21)	15.6% (10)	
1x per week		11.3% (8)	7.8% (5)	
2x per week		15.4% (11)	-	
3x per week		11.3% (8)	-	
4x per week		4.2% (3)	-	
5–6x per week		2.8% (2)	-	
Daily		-	-	

Variables expressed as percentage (n) or median (IQR), P-values calculated by Mann-Whitney U-test or chi-square test

### Associations of chronic high ED consumption and cardiological parameters

Regarding blood pressure and heart rate, the present study observed no differences in systolic and diastolic blood pressure or heart rate between participants with a chronic high ED consumption in comparison to the control group, neither in the unadjusted, nor in the adjusted models 2 and 3 (all *p* ≥ 0.05, Table 2). The present study observed no differences regarding the classification according to the German Hypertension League between adolescents with chronic high ED consumption compared to the control group (*p* = 0.54, Supplemental Table 2). Regarding electrocardiographic parameters, the present study did not reveal significant differences in PQ interval, QTc interval or QRS duration between both groups (Table 2).

**Table 2:** Unadjusted and adjusted differences in cardiovascular and electrocardiographic parameters according to chronic high ED consumption or control group

		Chronic high ED consumers (*n* = 97)	Control group (*n* = 160)	
	n	Geometric mean (95%-CI)	Geometric mean (95%-CI)	*p-value*
SBP [mmHg]				
Model 1	257	120.7 (118.3-123.2)	118.7 (116.9-120.6)	*0.20*
Model 2	228	123.8 (118.1-129.7)	122.6 (116.4–129.0)	*0.58*
Model 3	228	124.5 (118.7-130.5)	122.8 (116.7-129.3)	*0.46*
DBP [mmHg]				
Model 1	257	75.9 (74.2–77.6)	74.3 (73.0-75.6)	*0.15*
Model 2	228	76.0 (71.8–80.5)	75.8 (71.1–80.8)	*0.88*
Model 3	228	76.6 (72.3–81.2)	76.2 (71.5–81.1)	*0.79*
Heart rate [BPM]				
Model 1	257	75.7 (73.0-78.5)	73.5 (71.5–75.6)	*0.21*
Model 2	228	74.6 (68.2–81.5)	71.4 (64.7–78.7)	*0.20*
Model 3	228	74.8 (68.5–81.8)	71.9 (65.2–79.2)	*0.23*
PQ interval [ms]				
Model 1	255	138.1 (134.5-141.8)	139.5 (136.7-142.5)	*0.54*
Model 2	226	135.2 (126.0-145.1)	138.2 (127.9-149.4)	*0.42*
Model 3	226	134.9 (125.5-144.9)	138.0 (127.6-149.2)	*0.40*
QTc interval [ms]				
Model 1	256	361.0 (355.3-366.7)	364.4 (359.9-368.9)	*0.35*
Model 2	227	366.1 (351.9-380.9)	370.9 (355.1-387.5)	*0.39*
Model 3	227	365.7 (351.4-380.5)	369.9 (354.2-386.3)	*0.45*
QRS duration [ms]				
Model 1	256	86.9 (84.9–88.8)	87.1 (85.6–88.6)	*0.87*
Model 2	227	88.8 (84.7–93.1)	89.0 (84.5–93.8)	*0.90*
Model 3	227	89.4 (85.4–93.8)	89.0 (84.5–93.7)	*0.76*

*SBP* Systolic blood pressure, *DBP* diastolic blood pressure

Varying numbers of study participants in the cardiological parameters and/ or models are caused by missing parameter measurements and/or missing information of included confounders, Model 1: unadjusted (ANOVA); Model 2: adjusted for age, sex, physical activity, smoking status, smoking weed and alcohol consumption (ANCOVA), Model 3: additionally adjusted for school type (ANCOVA), Statistically significant after Bonferroni correction for multiple comparisons (threshold is *p* = 0.0002)

With regard to the medical reference values of PQ interval, QTc interval and QRS duration, almost all participants were within the normal range, and there were no significant differences between participants with chronically high ED consumption compared to the control group (all p ≥ 0.05, Supplemental Table 2). With regard to echocardiographic parameters the present study initially noticed that participants with a chronic high ED consumption had a thicker septum thickness and lower e’ septal in the unadjusted model 1 (both *p* = 0.04, Supplemental Table 3), although the geometric mean still lay within physiologically healthy range. However, after Bonferroni correction or adjustment for important confounders (model 2 and 3) the associations were no longer statistically significant. Likewise, there were no differences between the two study groups with regard to all other echocardiographic parameters (Supplemental Table 3). As shown in supplemental Table 2, no differences to medical reference values of echocardiological parameters have been noticed between participants with chronically high ED consumption compared to the control group (Supplemental Table 2).

In addition, when a potential cluster effect by school type was taken into account, no changes in the results regarding all cardiovascular parameters were observed (data not shown). Finally, sensitivity analysis (median-split analysis) revealed no differences for participants who consumed above the median (> 4.5 mg caffeine from EDs/kg BW/day) and for participants who consumed below the median (≤ 4.5 mg caffeine from EDs/kg BW/day) compared to the control group (Supplemental Table 4). Further, neither the investigation of the 10% highest ED consumers (≥ 8.64 mg caffeine from EDs/kg BW/day; Supplemental Table 5), nor the investigation of the consumption of ≥ 6 mg caffeine from EDs/kg BW/day (Supplemental Table 6) or the investigation of participants who reported having consumed EDs more than 2 years (Supplemental Table 7) altered the results. In addition, no significant changes in outcomes were detected investigating the total caffeine intake (ED and other caffeinated beverages; Supplemental Table 8). Here, it is important to emphasize that the sensitivity analyses included partly very low number of participants in the subgroups.

### Adverse effects after ED consumption

In addition to the parameter of the cardiological examination, the EDKAR-study also provides information on adverse effects after consumption of EDs. Upon inquiry half (49.5%, *n* = 46) of the 93 chronic high ED consumers (missing *n* = 4) reported adverse effects following ED consumption. The most commonly reported adverse effects (Table 3) in our study population were palpitations, poor sleep, headache and chest pressure.

**Table 3:** Reported adverse effects (multiple answers) of chronic high ED consumers who experienced adverse effects (*n* = 44) ^a^

Adverse effects (Multiple answers)	% (Number of mentions)
Palpitations	70.5% (31)
Poor sleep	43.2% (19)
Headache	40.9% (18)
Chest pressure	31.8% (14)
Gastrointestinal disorders	29.6% (13)
Dizziness	27.3% (12)
Perspiration	22.7% (10)
Lack of breath	20.5% (9)
Freezing, shivering	9.1% (4)
Anxiety attacks	4.5% (2)
Others	13.6% (6)

^a^ Missing data *n* = 2

### Selection bias

As depicted in supplemental Table 9, comprehensive examinations regarding possible selection bias between all invited participants to study phase 2 (no cardiological examination) (*n* = 187) with chronic high consumption of EDs compared to all invited and cardiologically examined participants with chronic high consumption of EDs (*n* = 97) showed that both groups did not differ in terms of gender, age, BMI, physical activity, alcohol consumption. With regard to a possible selection bias of the control group, there were significant differences in gender, school type and alcohol consumption between participants who were invited (*n* = 264) compared to participants who were invited and underwent the cardiological examination (*n* = 160). In fact, boys, vocational school pupils and participants with more regular alcohol consumption were more likely not to follow the invitation to study phase 2 and therefore were not examined at the Charité - Universitätsmedizin Berlin (Supplemental Table 9).

## Discussion

This is the first study investigating differences in blood pressure, heart rate, electrocardiographic and echocardiographic parameters in adolescents (aged 15–18 years) with a chronic high consumption of EDs (*n* = 97) in comparison to a control group (*n* = 160). While half of the chronic high ED consumers reported some adverse effects which they related to their ED consumption, the present study could not detect any statistically significant and/or clinically relevant differences in a variety of cardiological parameters. Notably participants with a chronically high ED consumption tended to adhere to a more risk-tolerant lifestyle, particularly in relation to an increased cardiovascular risk.

Scientific evidence investigating the consumption of EDs with regard to cardiovascular risks in adolescence is limited and restricted to an acute setting. So far there are no data on possible cardiovascular health effects of chronic high ED consumption. Only the EDUCATE-study and Mansour et al. from Israel have investigated the possible effects of EDs on blood pressure and heart rate or single electrocardiographic and echocardiographic parameters in children and adolescents [23–26, 28]. Furthermore, with regard to adults, cardiovascular risks have only been investigated in the context of acute ED consumption.

In the present study, chronic high ED consumers showed no significant differences in heart rate and SBP or DBP compared to the control group. Regarding heart rate, previous studies conducted in adults with acute ED consumption provide conflicting findings [10]. A systematic review with meta-analysis by Lasheras et al. based on prospective clinical studies (*n* = 43) summarized the contradictory study findings, showing 71% of the studies (only 38% significant) reported an increase in resting heart rate compared to baseline or placebo [37]. In contrast, 24% of the studies (only 18% significant) reported a decrease in heart rate [37]. In line with this, the EDUCATE-study, which was conducted in children and adolescents, observed no statistically significant difference in heart rate at any time point after ED consumption compared to a baseline or control group [24, 28]. On the other hand, referring to the same study population of the EDUCATE-study (different measurement methods), Mandilaras et al. found a significantly lower mean heart rate 120 min after ED consumption compared to the placebo group, while other time periods did not show significant differences in mean heart rate [26].

Regarding SBP and DBP, a systematic review with meta-analysis in adults noticed an increase in SBP and DBP after acute ED consumption [38]. In detail, Gualberto et al. observed an elevation of SBP and DBP for up to two hours after ED consumption, while more pronounced effects were seen on SBP at 60–80 min and DBP at 120 min [38]. Another review indicated that in general BP increases 30 min after ED consumption, reaching a maximum after 60–90 min before decreasing back to baseline after about two to four hours [10]. This is consistent with the pharmacokinetics of caffeine [10]. Likewise, the EDCUATE-study also demonstrated a significant temporary elevation of SBP and DBP in healthy children and teenagers after ED consumption [24]. In detail Oberhoffer et al. showed an initial increase in SBP one hour after ED consumption [24]. Two and four hours after ED consumption, a decrease in SBP was observed compared to peak SBP [24]. Accordingly, the point in time appears to have an important role in BP change after ED consumption [38], although Oberhoffer et al. observed that bodyweight-adjusted ED consumption during morning hours is associated with a significantly higher 24-h SBP and 24-h DBP in healthy children and adolescents [23].

In the present study, no significant differences in PQ interval, QTc interval and QRS duration were observed for chronic high ED consumers compared to the control group. According to a recent review, moderate acute ED consumption with a caffeine intake of up to 200 mg does not seem to affect the QTc interval in young healthy adults [10]. In connection with an ED consumption of 1 L, three out of four studies in this review showed a significant increase of the QTc interval [10]. Regarding PR interval, most studies noticed no changes in PR interval after ED consumption [13, 39–43], while Shah et al. noticed a reduction in PR interval, but in a clinically non-meaningful manner [11]. To the best of our knowledge, only Mandilaras et al. have investigated potential effect of ED consumption on electrocardiographic parameters in children and adolescents, showing QTc intervals were not affected by acute EDs consumption, PR interval and QRS duration have not been investigated [26].

The acute effects of consuming EDs on myocardial function, as determined by echocardiographic parameters have been investigated in a small number of studies in adults [34, 44, 45], and one study in children and adolescents [25]. Menci et al. noticed in young adults that acute ED consumption had an impact on left ventricular (LV) function and LV inotropy, showing an increase in global longitudinal strain, LVEF and MAPSE after ED consumption compared to baseline [34]. Another study suggested an increase in LV contractility, but observed an unchanged global left ventricular function (e.g. LVEF) after one-hour post ED consumption [44]. In contrast, a more recent study noticed that an acute consumption of EDs caused a significant increase in TAPSE with no effect on global longitudinal strain when compared to control drink, in addition with unaltered LV systolic function [45]. Regarding children and teenager, Oberhoffer et al. showed no differences between the ED consumption group and the placebo group according to conventional echocardiographic parameters of LV function (e.g. LVEF) [25]. However, the authors proposed when analysing LV efficiency parameters deducted through the generation of non-invasive LV pressure–volume loops, a significantly lower cardiac efficiency after the ED consumption [25].

Importantly, while the present study could not detect any statistically significant and/or clinically relevant differences in blood pressure, heart rate or electrocardiographic and echocardiographic parameters, previously reported potential acute impacts on the cardiovascular system are conceivable. A noticeable effect on the cardiovascular system is indirectly suggested by the most commonly reported adverse effects after ED consumption affecting the cardiovascular system i.e. palpitations and chest pain in our study population. In comparison to previous findings from other studies, fundamental methodological differences in study designs of the EDKAR-study may explain different study findings according to the investigated cardiological parameters. First, the EDKAR-study is not an intervention study, the participants were expected to follow their normal daily routine. This means no specifications were made as to whether and in what time frame the participants consume EDs before their cardiological examinations in order not to overestimate possible associations. Following the methodology of an epidemiological observational study, the EDKAR-study is not designed to investigate potential short-term changes in blood pressure or other parameters after direct consumption of EDs (intervention), but rather to investigate lasting effects of a chronic high ED consumption on the cardiovascular system. Based on the results of the EDKAR-study it appears that the cardiovascular system in young healthy participants (aged 15–18 years) to be sufficiently adaptable and resistant as to mitigate potential negative health impacts as the cardiological parameters of the chronic high ED consumers do not differ from the control group. In addition, a potential habituation effect might be also hypothesized [24]. Indeed, in the present EDKAR-study the participants were only categorized as chronic high ED consumers, when they had reported to drink EDs at least four days a week for at least a year and more than 3 mg caffeine from EDs/kg BW/day, thus some habitual effect can be assumed. The study by Mansour et al. also investigated the impact of EDs on blood pressure and heart rate, showing that heart rate and DBP were not significantly affected at any time point in both groups [28]. In contrast to the EDCUATE-study, the participants in the intervention group were students who regularly consumed EDs (consuming at least two cans weekly) [28]. They also showed higher SBP values after ED consumption at 15–30 min, one hour, and two hours after drinking, compared to the control group [28].

Although, the present study revealed no concerning results with regard to a variety of cardiological parameters, yet EDs should nevertheless be consumed with due care. Indeed, previous studies and case reports suggest that (acute) ED consumption in children and adolescents may be associated with adverse effects on cardiovascular health, including case reports with sometimes fatal outcome [29]. Particular caution is required in adolescents with risk factors, such as the presence of pre-existing conditions or combination with potentially triggering factors (e.g. party drugs, alcohol or physical activity [23, 29, 37, 46]). It was noted that a relevant proportion of chronic high ED consumers in the present study seem to consume EDs in combination with alcohol. A present review of case reports of young adults indicate the simultaneous consumption of EDs and alcohol may be associated with an increased correlation for severe cardiovascular events, albeit without proof of causality [46]. Likewise, this study revealed that chronic high ED consumers had a considerably higher intake of alcohol, were more likely to smoke and/or smoke weed, as well as having shortened sleep patterns. In line, a present systematic review conducted in young adults observed that ED consumption was frequently associated with alcohol use and smoking [47]. Regarding alcohol it has been proposed that the earlier the alcohol consumption, the greater the risk of adolescents becoming excessive consumers of alcohol throughout life [48], which increased the further risk of cardiovascular diseases [49]. Moreover, smoking is considered as a well-known risk factor for the development of cardiovascular diseases. Referring to adolescents, a recently published prospective cohort study showed that youth smoking beginning in early childhood, were associated with adult cardiovascular events [50]. Also sufficient sleep is considered important for optimal health promotion [51]. For adolescents aged 13 to 18 years, a sleep duration of between 8 and 10 h per 24 h is recommended [51]. In line with our study, a previous study in young adults [52] and minors [23, 53] noticed that acute ED consumption is associated with a shorter sleep duration, which might additionally increase cardiovascular risk [54]. However, if and to what degree the chronic consumption of EDs triggers this behaviour or vice versa or to which degree these factors are possibly influenced by a more risk-tolerant attitude cannot be clarified and is beyond the scope of this study.

Ideally children and adolescents should be informed about potential ED-associated risk factors to allow for an informed choice [23]. It has been shown that participants with a greater awareness of the ingredients and potential adverse effects of EDs consumed less EDs [28]. It should also be noticed that chronic ED consumption may lead to sugar metabolism disorders and obesity due to their high sugar content, which might additionally increase cardiovascular morbidity in long term manner [23, 29]. Given that the use of EDs is particularly popular among minors, further research is urgently needed in this vulnerable group.

Strengths of our EDKAR-study include the cardiological examination, which carried out in a blinded manner with regard to group affiliation (chronic high ED consumers vs. control group). Moreover, as part of study phase 1, comprehensive information on important lifestyle factors, as well as the frequency/consumption of EDs and other caffeinated beverages were collected via an online questionnaire. Thus, data from important confounders were determined and taken into account in the statistical models. Although, the planned age- and sex-matched enrolment in study phase 2 with matching factor 1:2 had to be waived, no significant differences in sex and age in the final study population of both groups were observed. Moreover, some limitations of our study deserve to be mentioned. Due to the requirements of the Berlin administration, the study team at the BfR were not permitted to have any contact details of the adolescents during study phase 1. As a result, in study phase 2 we had to use allocation lists in the classes and were dependent on the cooperation of the teaching staff in the schools i.e. correct handover of invitations to study phase 2. Thus, the study team had no possibility of directly contacting invited pupils and motivating them to take part in study phase 2 (cardiological examination). Nevertheless, the present study achieved satisfactory response rates in both groups (chronic high ED consumption: 34.4%, control group: 37.7%). In addition, no relevant selection bias of all invited pupils (no response to the invitation) compared to cardiological examined participants has been observed. Further seen as limitation, the pupils were recruited exclusively in Berlin schools, so that a generalization of the results, e.g. for students in rural areas or other cities, cannot be made. Finally, this is a cross-sectional study not allowing causal inferences.

Based on comprehensive cardiological examination the present EDKAR-study could not detect any statistically significant and/or clinically relevant differences in a variety of cardiological parameters. However, half of the chronic high ED consumers reported having experienced adverse effects after consuming EDs. Altogether the data may indicate the that the adolescent cardiovascular system is sufficiently adaptable to mitigate respective potential adverse effects. It should be noted, that the study design does not allow for conclusions about potential risks at a later age, particularly for individuals subject to other risk factors such as smoking, high alcohol consumption or pre-existing illnesses, as well as a simultaneous consumption of EDs with other substances such as party drugs.

## Supplementary Information

Below is the link to the electronic supplementary material.

Supplementary Material 1

## Data Availability

The data used in the present study will not be made publicly available, but they are accessible by contacting the corresponding author.
