# Supplementary material for: Chronic high consumption of energy drinks and cardiovascular risk in adolescents—results of the EDKAR-study
Source: Eur J Epidemiol. 2025 Aug 23;40(11):1355–68. doi: 10.1007/s10654-025-01292-z (PMC12696085; doi:10.1007/s10654-025-01292-z)
Supplement: Supplementary file 1 — Supplementary Material 1 [file 10654_2025_1292_MOESM1_ESM.docx]

**Supplemental Figure 1.** Directed acyclic graphs (DAG) of the potential confounders to estimate the minimal sufficient adjustment set for the total effect of ED consumption on cardiological parameter


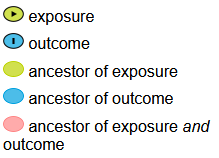
**
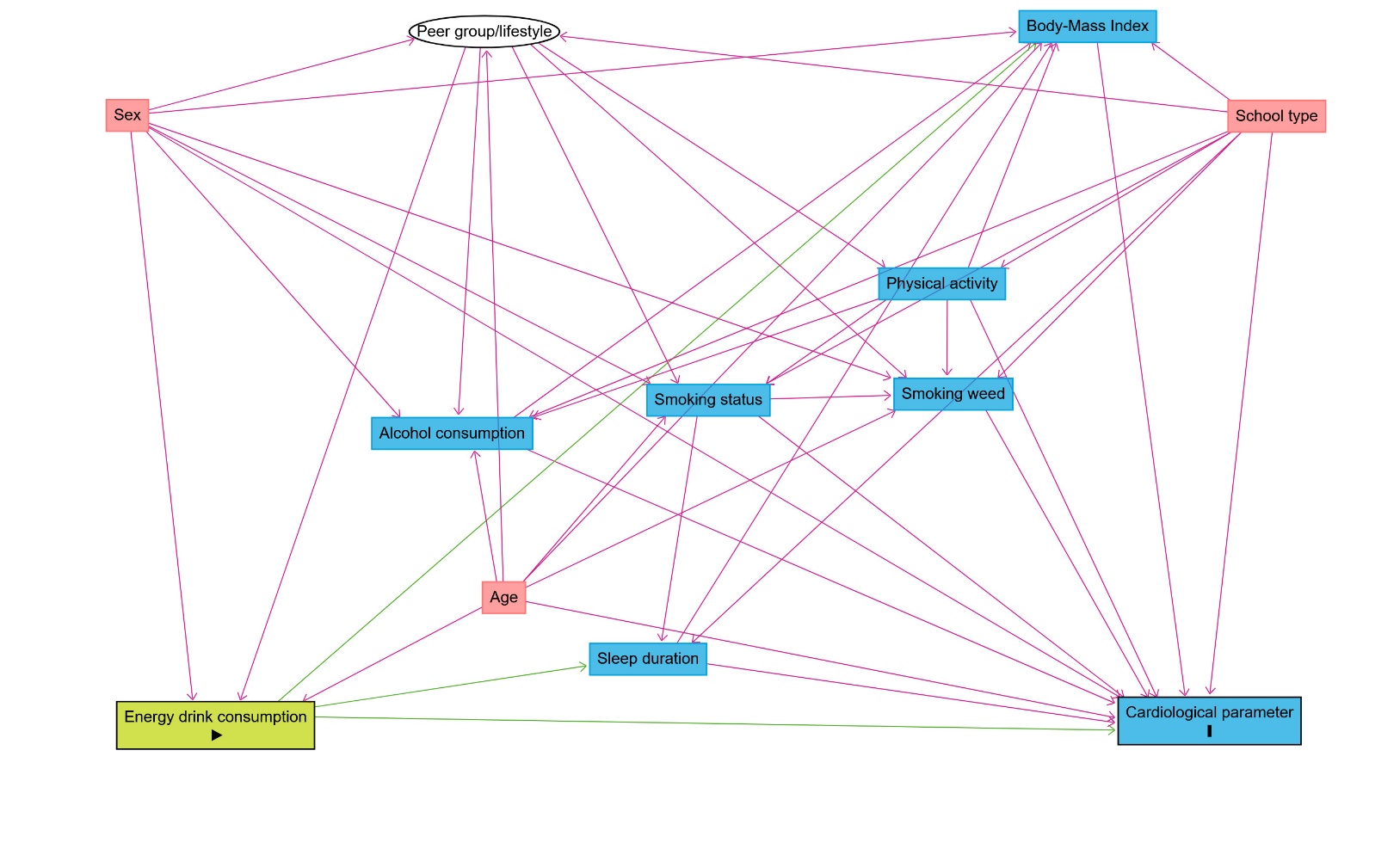
**

All arrows lying on open biasing paths are highlighted in magenta and all arrows lying on open causal paths in green.

**Supplemental Table 1**. Sleep duration on the weekends between chronic high ED consumers (n = 97) compared to control group (n = 158)

|  | **Chronic high ED consumers (n=97)** | **Control group (n=158)** | *p-value* |
| --- | --- | --- | --- |
| Sleep duration during weekends |  |  | *0.005* |
| < 5 h | 5.2% (5) | 0% (0) |  |
| 5-6 h | 7.2% (7) | 1.9% (3) |  |
| 6-7 h | 10.3% (10) | 5.7% (9) |  |
| 7-8 h | 10.3% (10) | 15.2% (24) |  |
| 8-9 h | 23.7% (23) | 32.9% (52) |  |
| 9-10 h | 20.6% (20) | 27.2% (43) |  |
| 10-11 h | 14.4% (14) | 13.3% (21) |  |
| >11 h | 8.3% (8) | 3.8% (6) |  |

P-values calculated by chi-square test

**Supplemental Table 2**. Investigation of differences in blood pressure, electrocardiographic and echocardiographic parameters between chronic high ED consumers (n = 97) compared the control group (n = 160) according to classifications of reference groups

|  |  | **Chronic high ED consumers (n=97)** | **Control group (n=160)** |  |
| --- | --- | --- | --- | --- |
|  | Reference groups | **% (n)** | **% (n)** | *p-Value* |
| Blood pressure |  |  |  | *0.54* |
| Optimal | SBP mmHg < 120 and DBP < 80 mmHg | 44.3% (43) | 53.1% (85) |  |
| Normal | SBP mmHg 120-129 and/ or DBP 80-84 mmHg | 33.0% (32) | 25.6% (41) |  |
| High normal | SBP mmHg 130-139 and/ or DBP 85-89 mmHg | 17.5% (17) | 16.9% (27) |  |
| Hypertension | SBP mmHg ≥ 140 and/ or DBP ≥ 90 mmHg | 5.2% (5) | 4.4% (7) |  |
| PQ interval [ms] |  |  |  | *0.10* |
| Normal | 120-200 ms | 92.8% (90) | 86.1% (136) |  |
| Shortened | < 120 ms | 7.2% (7) | 13.9% (22) |  |
| Extended | > 200 ms | - | - |  |
| QTc interval [ms] |  |  |  | *0.43* |
| Normal | Male < 450 ms / Female < 470 ms | 100% (97) | 99.4 (158) |  |
| Outside normal reference | Male ≥ 450 ms / Female ≥ 470 ms | - | 0.6 (1) |  |
| QRS duration [ms] |  |  |  | *0.61* |
| Normal | < 100 ms | 90.7% (88) | 88.7% (141) |  |
| Incomplete bundle branch block | 100-120 ms | 9.3% (9) | 11.3% (18) |  |
| Complete bundle branch block | > 120 ms | - | - |  |
| LVEF [%] |  |  |  | *0.52* |
| Normal | Male ≥ 52% / Female ≥ 54% | 96.9% (93) | 98.1% (157) |  |
| Slightly reduced | Male 41-51% / Female 41-53% | 3.1% (3) | 1.9% (3) |  |
| Moderately reduced | Male 30-40% / Female 30-40% | - | - |  |
| Highly reduced | Male < 30% / Female <30% | - | - |  |
| LVEDd [mm] |  |  |  | *0.27* |
| Normal | Male ≤ 58 mm / Female ≤ 52 mm | 100.0% (96) | 98.7% (157) |  |
| Slightly reduced | Male 59-63 mm / Female 53-56 mm | - | - |  |
| Moderately reduced | Male 64-68 mm / Female 57-61 mm | - | - |  |
| Highly reduced | Male > 68 mm / Female > 61 mm | - | 1.3% (2) |  |
| IVSd [mm] |  |  |  | *0.18* |
| Normal | Male ≤ 10 mm / Female ≤ 9 mm | 81.3% (78) | 87.4% (139) |  |
| Slightly reduced | Male 11-13 mm / Female 10-12 mm | 18.8% (18) | 12.6% (20) |  |
| Moderately reduced | Male 14-16 mm / Female 13-15 mm | - | - |  |
| Highly reduced | Male > 16 mm / Female > 15 mm | - | - |  |
| PWED [mm] |  |  |  | *0.88* |
| Normal | Male ≤ 10 mm / Female ≤ 9 mm | 95.8% (92) | 96.2% (153) |  |
| Slightly reduced | Male 11-13 mm / Female 10-12 mm | 4.2% (4) | 3.8% (6) |  |
| Moderately reduced | Male 14-16 mm / Female 13-15 mm | - | - |  |
| Highly reduced | Male > 16 mm / Female > 15 mm | - | - |  |
| LVMI [g/m^2^] |  |  |  | *0.33* |
| Normal | Male ≤ 102 g/m^2^ / Female ≤ 88 g/m^2^ | 92.7% (89) | 95.6% (152) |  |
| Hypertrophy | Male > 102 g/m^2^ / Female > 88 g/m^2^ | 7.3% (7) | 4.4% (7) |  |
| LAVI [ml/ml^2^] |  |  |  | *0.20* |
| Normal | < 34 ml/ml^2^ | 98.9% (92) | 100.0% (154) |  |
| Outside normal reference | ≥ 34 ml/ml^2^ | 1.1% (1) | - |  |
| TAPSE [mm] |  |  |  | *0.72* |
| Normal | ≥ 17 mm | 98.9% (95) | 99.4% (157) |  |
| Outside normal reference | < 17 mm | 1.0% (1) | 0.6% (1) |  |
| MAPSE [mm] |  |  |  |  |
| Normal | ≥ 10 mm | 100.0% (96) | 100.0% (158) |  |
| Outside normal reference | < 10 mm | - | - |  |
| E/A |  |  |  | *0.17* |
| Normal | 0.8 to 2.0 | 75.8% (72) | 67.72% (107) |  |
| Outside normal reference | <0.8 or > 2.0 | 24.2% (23) | 32.3% (51) |  |
| e’ lateral [cm/s] |  |  |  | *0.70* |
| Normal | > 10 m/s | 98.9% (90) | 99.4% (154) |  |
| Outside normal reference | ≤ 10 m/s | 1.1% (1) | 0.6% (1) |  |
| e’ septal [cm/s] |  |  |  |  |
| Normal | > 7 m/s | 100.0% (92) | 100.0% (155) |  |
| Outside normal reference | ≤ 7 m/s | - | - |  |
| E / e’ mean |  |  |  |  |
| Normal | < 14 | 100.0% (90) | 100.0% (152) |  |
| Outside normal reference | ≥ 14 | - | - |  |
| Global longitudinal strain (%) |  |  |  | *0.78* |
| Normal | ≤ -16% | 91.8% (78) | 92.8% (129) |  |
| Dysfunction | > -16% | 8.2% (7) | 7.2% (10) |  |

P-values calculated by chi-square test, Abbreviations: SBP: Systolic blood pressure, DBP: diastolic blood pressure, LVEF: Left ventricular ejection fraction, LVEDd: Left ventricular end-diastolic diameter, IVSd: Interventricular septum thickness , PWED: Posterior wall thickness, LVMI: Left ventricular mass index, LAVES (4CH): Left atrial volume four-chambers, LAVES (2CH): Left atrial volume two-chambers, LAVES mean: Left atrial volume mean from LAVES (4CH) and LAVES (2CH), LAVI: Left atrial volume index, TAPSE: Tricuspid annular plane systolic excursion, MAPSE: Mitral annular plane systolic excursion

**Supplemental Table 3.** Differences in echocardiographic parameters according to chronic high ED consumption or control group

|  |  | Chronic high consumers (n=97) | Control group  (n=160) |  |
| --- | --- | --- | --- | --- |
|  | n | Geometric mean (95%-CI) | Geometric mean (95%-CI) | *p-value* |
| LVEF [%] |  |  |  |  |
| Model 1 | 256 | 60.4 (59.5-61.2) | 60.9 (60.3-61.5) | *0.31* |
| Model 2 | 227 | 59.9 (57.9-62.0) | 61.1 (58.8-63.5) | *0.14* |
| Model 3 | 227 | 60.0 (57.9-62.1) | 61.2 (58.9-63.5) | *0.14* |
| LVEDd [mm] |  |  |  |  |
| Model 1 | 255 | 44.7 (43.6-45.9) | 44.4 (43.5-45.2) | *0.59* |
| Model 2 | 226 | 45.1 (42.4-47.9) | 45.4 (42.4-48.6) | *0.22* |
| Model 3 | 216 | 45.6 (42.9-48.5) | 45.5 (42.6-48.7) | *0.93* |
| IVSd [mm] |  |  |  |  |
| Model 1 | 255 | 8.69 (8.36-9.03) | 8.25 (8.00-8.50) | *0.04* |
| Model 2 | 226 | 9.20 (8.40-10.1) | 9.01 (8.15-9.96) | *0.55* |
| Model 3 | 226 | 9.26 (8.44-10.2) | 9.01 (8.15-9.97) | *0.44* |
| PWED [mm] |  |  |  |  |
| Model 1 | 255 | 7.51 (7.23-7.81) | 7.65 (7.42-7.88) | *0.47* |
| Model 2 | 226 | 7.79 (7.07-8.58) | 8.12 (7.30-9.03) | *0.25* |
| Model 3 | 226 | 7.86 (7.13-8.67) | 8.15 (7.32-9.06) | *0.34* |
| LVMI [g/m^2^] |  |  |  |  |
| Model 1 | 255 | 69.0 (65.6-72.6) | 66.5 (63.9-69.1) | *0.24* |
| Model 2 | 226 | 76.4 (67.9-86.0) | 77.5 (68.0-88.3) | *0.76* |
| Model 3 | 226 | 78.5 (69.8-88.3) | 77.8 (68.4-88.4) | *0.83* |
| LAVES (4CH) [ml] |  |  |  |  |
| Model 1 | 250 | 32.1 (29.8-34.5) | 31.6 (29.8-33.4) | *0.73* |
| Model 2 | 221 | 35.0 (29.4-41.7) | 32.5 (26.8-39.4) | *0.26* |
| Model 3 | 221 | 35.9 (30.1-42.7) | 32.4 (26.8-39.3) | *0.14* |
| LAVES (2CH) [ml] |  |  |  |  |
| Model 1 | 248 | 37.7 (35.0-40.5) | 35.9 (33.9-37.9) | *0.29* |
| Model 2 | 219 | 35.8 (30.0-42.6) | 34.4 (28.3-41.8) | *0.58* |
| Model 3 | 219 | 35.6 (29.8-42.5) | 34.1 (28.1-41.4) | *0.54* |
| LAVES mean [ml] | | | | |
| Model 1 | 248 | 35.2 (33.0-37.6) | 34.2 (32.5-36.0) | *0.50* |
| Model 2 | 219 | 35.8 (30.7-41.7) | 34.1 (28.8-40.4) | *0.41* |
| Model 3 | 219 | 36.1 (31.0-42.1) | 33.9 (28.7-40.1) | *0.29* |
| LAVI [ml/ml^2^] |  |  |  |  |
| Model 1 | 247 | 20.8 (19.7-21.9) | 20.7 (19.9-21.6) | *0.93* |
| Model 2 | 218 | 20.4 (17.8-23.4) | 20.4 (17.6-23.7) | *0.98* |
| Model 3 | 218 | 20.6 (18.0-23.5) | 20.2 (17.5-23.4) | *0.75* |
| TAPSE [mm] |  |  |  |  |
| Model 1 | 254 | 22.7 (22.0-23.4) | 22.8 (22.3-23.3) | *0.87* |
| Model 2 | 225 | 23.2 (21.5-25.1) | 23.0 (21.1-25.1) | *0.78* |
| Model 3 | 225 | 23.2 (21.5-25.1) | 23.1 (21.2-25.1) | *0.82* |
| MAPSE [mm] |  |  |  |  |
| Model 1 | 254 | 15.9 (15.5-16.4) | 15.8 (15.4-16.1) | *0.57* |
| Model 2 | 225 | 16.0 (14.9-17.2) | 15.7 (14.5-17.0) | *0.49* |
| Model 3 | 225 | 16.1 (14.9-17.3) | 15.7 (14.5-17.0) | *0.44* |
| E-Wave [cm/s] |  |  |  |  |
| Model 1 | 253 | 85.4 (82.5-88.5) | 88.9 (86.5-91.3) | *0.08* |
| Model 2 | 224 | 86.4 (79.0-94.5) | 90.1 (81.6-99.5) | *0.22* |
| Model 3 | 224 | 86.1 (78.6-94.2) | 90.3 (81.8-99.7) | *0.16* |
| A-Wave [cm/s] |  |  |  |  |
| Model 1 | 253 | 49.7 (47.4-52.1) | 49.4 (47.6-51.3) | *0.84* |
| Model 2 | 224 | 52.2 (46.3-58.7) | 52.7 (46.2-60.1) | *0.83* |
| Model 3 | 224 | 52.4 (46.5-59.1) | 53.1 (46.6-60.4) | *0.78* |
| E / A |  |  |  |  |
| Model 1 | 253 | 1.72 (1.63-1.81) | 1.80 (1.73-1.87) | *0.17* |
| Model 2 | 224 | 1.66 (1.45-1.89) | 1.71 (1.48-1.97) | *0.51* |
| Model 3 | 224 | 1.64 (1.44-1.87) | 1.70 (1.47-1.97) | *0.48* |
| e‘ lateral [cm/s] |  |  |  |  |
| Model 1 | 246 | 17.6 (17.0-18.3) | 17.8 (17.3-18.3) | *0.65* |
| Model 2 | 218 | 17.6 (16.1-19.7) | 17.8 (16.1-19.7) | *0.79* |
| Model 3 | 218 | 17.6 (16.1-19.2) | 17.8 (16.1-19.7) | *0.82* |
| e‘ septal [cm/s] |  |  |  |  |
| Model 1 | 247 | 13.7 (13.3-14.2) | 14.3 (14.0-14.7) | *0.04* |
| Model 2 | 218 | 13.3 (12.2-14.5) | 13.9 (12.6-15.3) | *0.18* |
| Model 3 | 218 | 13.1 (12.0-14.3) | 13.8 (12.6-15.2) | *0.10* |
| e‘ mean [cm/s] |  |  |  |  |
| Model 1 | 245 | 15.7 (15.2-16.2) | 16.1 (15.8-16.5) | *0.16* |
| Model 2 | 217 | 15.5 (14.4-16.7) | 15.9 (14.7-17.3) | *0.37* |
| Model 3 | 217 | 15.4 (14.3-16.6) | 15.9 (14.6-17.3) | *0.31* |
| E / e’ lateral |  |  |  |  |
| Model 1 | 243 | 4.86 (4.65-5.08) | 5.01 (4.85-5.19) | *0.27* |
| Model 2 | 215 | 4.91 (4.38-5.49) | 5.14 (4.54-5.82) | *0.28* |
| Model 3 | 215 | 4.88 (4.35-5.47) | 5.16 (4.55-5.84) | *0.22* |
| E / e’ septal |  |  |  |  |
| Model 1 | 244 | 6.21 (5.95-6.50) | 6.23 (6.02-6.45) | *0.93* |
| Model 2 | 215 | 6.49 (5.78-7.28) | 6.58 (5.79-7.48) | *0.75* |
| Model 3 | 215 | 6.53 (5.82-7.33) | 6.62 (5.83-7.51) | *0.77* |
| E / e’ mean |  |  |  |  |
| Model 1 | 242 | 5.44 (5.23-5.67) | 5.54 (5.36-5.71) | *0.53* |
| Model 2 | 214 | 5.57 (5.02-6.18) | 5.75 (5.13-6.45) | *0.42* |
| Model 3 | 314 | 5.57 (5.02-6.18) | 5.78 (5.15-6.47) | *0.37* |
| Global longitudinal strain [%] | | | | |
| Model 1 | 224 | -19.8 (-21.1 to -18.3) | -20.2 (-21.2 to -19.1) | *0.63* |
| Model 2 | 200 | -18.5 (-22.0 to -13.3) | -20.2 (-23.4 to -15.3) | *0.27* |
| Model 3 | 200 | -18.5 (-22.0 to -13.2) | -20.2 (-23.4 to -15.2) | *0.29* |

Expressed as geometric mean (95%-CI), Varying numbers of study participants in the cardiological parameters and/ or models are caused by missing parameter measurements and/or missing information of included confounders, Model 1: unadjusted (ANOVA); Model 2: adjusted for age, sex, physical activity, smoking status, smoking weed and alcohol consumption (ANCOVA), Model 3: additionally adjusted for school type (ANCOVA), Statistically significant after Bonferroni correction for multiple comparisons (threshold is p = 0.0002). Abbreviations: LVEF: Left ventricular ejection fraction, LVEDd: Left ventricular end-diastolic diameter, IVSd: Interventricular septum thickness , PWED: Posterior wall thickness, LVMI: Left ventricular mass index, LAVES (4CH): Left atrial volume four-chambers, LAVES (2CH): Left atrial volume two-chambers, LAVES mean: Left atrial volume mean from LAVES (4CH) and LAVES (2CH), LAVI: Left atrial volume index, TAPSE: Tricuspid annular plane systolic excursion, MAPSE: Mitral annular plane systolic excursion

**Supplemental Table 4.** Stratified analyses (median-split) investigating differences in cardiological parameters between participants with ED consumption ≤ 4.5 mg caffeine from EDs/kg BW/day or > 4.5 caffeine from EDs/kg BW/day compared to the control group

|  |  | **Chronic high ED consumption** | | | **Control group (n=160)** | |  |  |
| --- | --- | --- | --- | --- | --- | --- | --- | --- |
|  |  | ≤ 4.5 mg caffeine EDs /kg BW/ day (n=47) |  | > 4.5 mg caffeine EDs /kg BW/ day (n=50) |  | No ED consumption |  |  |
|  | n | Median (Q1-Q3) | n | Median (Q1-Q3) | n | Median (Q1-Q3) | *p-Values ^a^* | *p-Values ^b^* |
| SBP [mmHg] | 47 | 122.0 (113.5- 130.5) | 50 | 120.8 (113.5- 127.0) | 160 | 118.3 (110.3-128.3) | *0.10* | *0.48* |
| DBP [mmHg] | 47 | 77.5 (71.0-84.0) | 50 | 73.8 (68.0-82.0) | 160 | 74.0 (68.3-80.5) | *0.08* | *0.75* |
| Heart rate [BPM] | 47 | 75.0 (66.0-87.0) | 50 | 75.0 (69.0-85.0) | 160 | 73.0 (64.0-84.0) | *0.47* | *0.35* |
| **Electrocardiographic parameters** | |  |  |  |  |  |  |  |
| PQ interval [ms] | 47 | 140.0 (126.0-148.0) | 50 | 140.0 (130.0-152.0) | 159 | 140.0 (130.0-150.0) | *0.49* | *0.81* |
| QTc interval [ms] | 47 | 358.0 (340.0-382.0) | 50 | 362.0 (342.0-378.0) | 159 | 364.0 (344.0-384.0) | *0.33* | *0.50* |
| QRS duration [ms] | 47 | 84.0 (80.0-90.0) | 50 | 90.0 (84.0-96.0) | 159 | 88.0 (82.0-94.0) | *0.08* | *0.15* |
| **Echocardiographic parameters** | | |  |  |  |  |  |  |
| LVEF [%] | 47 | 61.0 (58.0-64.0) | 49 | 60.0 (56.0-64.0) | 160 | 61.0 (58.0-64.0) | *0.97* | *0.26* |
| LVEDd [mm] | 47 | 44.0 (42.0-48.0) | 49 | 44.0 (42.0-48.0) | 159 | 44.0 (41.0-48.0) | *0.49* | *0.57* |
| IVSd [mm] | 47 | 9.00 (8.00-10.0) | 49 | 9.00 (7.90-10.0) | 159 | 8.00 (7.00-9.00) | *0.28* | *0.07* |
| PWED [mm] | 47 | 7.00 (6.00-8.00) | 49 | 8.00 (7.00-9.00) | 159 | 8.00 (7.00-9.00) | *0.18* | *0.99* |
| LVMI [g/m^2^] | 47 | 66.0 (56.0-79.0) | 49 | 71.5 (59.0-89.8) | 159 | 68.3 (56.4-80.3) | *0.85* | *0.17* |
| LAVES (4CH) [ml] | 46 | 33.9 (26.0-44.0) | 49 | 31.9 (25.0-39.0) | 155 | 31.6 (25.0-40.8) | *0.60* | *0.94* |
| LAVES (2CH) [ml] | 44 | 38.9 (27.0-49.0) | 49 | 37.1 (30.0-48.4) | 155 | 36.0 (28.0-46.4) | *0.42* | *0.47* |
| LAVES mean [ml] | 44 | 35.8 (29.7-44.2) | 49 | 36.0 (27.3-42.5) | 155 | 34.5 (28.0-43.0) | *0.52* | *0.82* |
| LAVI [ml/ml^2^] | 44 | 21.0 (17.0-24.2) | 49 | 22.0 (17.1-25.5) | 154 | 21.0 (18.0-26.0) | *0.64* | *0.83* |
| TAPSE [mm] | 47 | 23.0 (20.0-25.0) | 48 | 23.0 (20.0-26.0) | 158 | 23.0 (21.0-25.0) | *0.95* | *0.84* |
| MAPSE [mm] | 47 | 16.0 (14.2-17.8) | 48 | 16.0 (14.4-17.9) | 158 | 16.0 (14.0-17.0) | *0.43* | *0.95* |
| E-Wave [cm/s] | 47 | 87.0 (77.0-98.0) | 48 | 84.0 (77.0-91.5) | 158 | 90.0 (80.0-99.0) | *0.47* | *0.03* |
| A-Wave [cm/s] | 47 | 50.0 (43.0-59.0) | 48 | 49.5 (41.5-59.0) | 158 | 50.0 (41.0-58.0) | *0.46* | *0.96* |
| E / A | 47 | 1.66 (1.44-2.00) | 48 | 1.63 (1.47-2.03) | 158 | 1.80 (1.50-2.20) | *0.23* | *0.20* |
| e‘ lateral [cm/s] | 47 | 18.0 (16.0-20.0) | 44 | 17.5 (15.0-19.7) | 155 | 18.0 (16.0-20.4) | *0.96* | *0.41* |
| e‘ septal [cm/s] | 47 | 14.0 (13.0-15.0) | 45 | 14.0 (13.0-15.0) | 155 | 14.0 (13.0-16.0) | *0.18* | *0.06* |
| e‘ mean [cm/s] | 47 | 16.0 (14.5-17.5) | 44 | 15.5 (14.3-17.8) | 154 | 16.0 (14.5-18.0) | *0.61* | *0.17* |
| E / e’ lateral | 47 | 4.79 (4.40-5.40) | 43 | 5.06 (4.10-5.63) | 153 | 4.83 (4.29-5.91) | *0.49* | *0.47* |
| E / e’ septal | 47 | 6.23 (5.50-6.94) | 44 | 5.93 (5.16-7.09) | 153 | 6.20 (5.40-7.27) | *0.77* | *0.47* |
| E / e’ mean | 47 | 5.47 (4.79-6.07) | 43 | 5.38 (4.65-6.22) | 152 | 5.51 (4.87-6.36) | *0.67* | *0.53* |
| Global longitudinal strain [%] | 40 | -20.1 (-21.9 to -18.0) | 45 | -20.0 (-21.1 to -18.0) | 139 | -20.0 (-22.0 to -18.0) | *0.94* | *0.50* |

Varying numbers of study participants in the cardiological parameters are caused by missing parameter measurements,^a^ Comparison between chronic high consumers with ≤ 4.5 mg caffein from ED/ kg BW/ day in comparison to control group, ^b^ Comparison between chronic high consumers with > 4.5 mg caffein from ED/ kg BW/ day in comparison to control group, P-values calculated by Mann-Whitney U-test, Statistically significant after Bonferroni correction for multiple comparisons (threshold is p = 0.0002), SBP: Systolic blood pressure, DBP: Diastolic blood pressure, Abbreviations: SBP: Systolic blood pressure, DBP: diastolic blood pressure, LVEF: Leftventricular ejection fraction, LVEDd: Left ventricular end-diastolic diameter, IVSd: Interventricular septum thickness , PWED: Posterior wall thickness, LVMI: Left ventricular mass index, LAVES (4CH): Left atrial volume four-chambers, LAVES (2CH): Left atrial volume two-chambers, LAVES mean: Left atrial volume mean from LAVES (4CH) and LAVES (2CH), LAVI: Left atrial volume index, TAPSE: Tricuspid annular plane systolic excursion, MAPSE: Mitral annular plane systolic excursion

**Supplemental Table 5**. Investigation of differences in blood pressure, heart rate, electrocardiographic and echocardiographic parameters between participants with the 10% highest caffeine intake from EDs (n = 10) compared to the control group (n = 160)

|  |  | **Chronic high ED consumers** |  | **Control group (n=160)** |  |
| --- | --- | --- | --- | --- | --- |
|  |  | ≥ 8.64 mg caffeine from EDs /kg BW/day (n=10) |  | No ED consumption |  |
|  | n | Median (Q1-Q3) | n | Median (Q1-Q3) | *p-Values* |
| SBP [mmHg] | 10 | 120.8 (109.0- 125.5) | 160 | 118.3 (110.3-128.3) | *0.97* |
| DBP [mmHg] | 10 | 79.0 (68.0-81.5) | 160 | 74.0 (68.3-80.5) | *0.59* |
| Heart rate [BPM] | 10 | 72.5 (67.0-77.0) | 160 | 73.0 (64.0-84.0) | *0.90* |
| **Electrocardiographic parameters** |  |  |  |  |  |
| PQ interval [ms] | 10 | 139.0 (130.0-154.0) | 159 | 140.0 (130.0-150.0) | *0.97* |
| QTc interval [ms] | 10 | 361.0 (344.0-378.0) | 159 | 364.0 (344.0-384.0) | *0.89* |
| QRS duration [ms] | 10 | 87.0 (86.0-92.0) | 159 | 88.0 (82.0-94.0) | *0.21* |
| **Echocardiographic parameters** |  |  |  |  |  |
| LVEF [%] | 10 | 61.5 (56.0-63.0) | 160 | 61.0 (58.0-64.0) | *0.48* |
| LVEDd [mm] | 10 | 43.0 (42.0-48.0) | 159 | 44.0 (41.0-48.0) | *0.60* |
| IVSd [mm] | 10 | 9.00 (7.90-9.90) | 159 | 8.00 (7.00-9.00) | *0.52* |
| PWED [mm] | 10 | 7.00 (6.10-7.60) | 159 | 8.00 (7.00-9.00) | *0.39* |
| LVMI [g/m^2^] | 10 | 68.3 (63.1-80.9) | 159 | 68.3 (56.4-80.3) | *0.20* |
| LAVES (4CH) [ml] | 10 | 36.0 (30.0-39.0) | 155 | 31.6 (25.0-40.8) | *0.71* |
| LAVES (2CH) [ml] | 10 | 44.0 (37.1-52.0) | 155 | 36.0 (28.0-46.4) | *0.92* |
| LAVES mean [ml] | 10 | 38.8 (36.0-45.5) | 155 | 34.5 (28.0-43.0) | *0.75* |
| LAVI [ml/ml^2^] | 10 | 24.3 (22.0-25.5) | 154 | 21.0 (18.0-26.0) | *0.84* |
| TAPSE [mm] | 10 | 20.5 (18.1-23.0) | 158 | 23.0 (21.0-25.0) | *0.73* |
| MAPSE [mm] | 10 | 15.0 (14.0-16.0) | 158 | 16.0 (14.0-17.0) | *0.83* |
| E-Wave [cm/s] | 10 | 81.5 (76.0-88.0) | 158 | 90.0 (80.0-99.0) | *0.25* |
| A-Wave [cm/s] | 10 | 43.0 (40.0-51.0) | 158 | 50.0 (41.0-58.0) | *0.54* |
| E / A | 10 | 1.78 (1.58-2.10) | 158 | 1.80 (1.50-2.20) | *0.16* |
| e‘ lateral [cm/s] | 10 | 18.0 (16.0-19.2) | 155 | 18.0 (16.0-20.4) | *0.58* |
| e‘ septal [cm/s] | 10 | 15.0 (13.0-16.0) | 155 | 14.0 (13.0-16.0) | *0.22* |
| e‘ mean [cm/s] | 10 | 15.8 (15.4-17.5) | 154 | 16.0 (14.5-18.0) | *0.36* |
| E / e’ lateral | 10 | 4.49 (3.92-5.33) | 153 | 4.83 (4.29-5.91) | *0.53* |
| E / e’ septal | 10 | 5.50 (5.00-5.94) | 153 | 6.20 (5.40-7.27) | *0.86* |
| E / e’ mean | 10 | 4.86 (4.43-5.94) | 152 | 5.51 (4.87-6.36) | *0.64* |
| Global longitudinal strain [%] | 10 | -20.7 (-22.0 to -19.0) | 139 | -20.0 (-22.0 to -18.0) | *0.96* |

Varying numbers of study participants in the cardiological parameters are caused by missing parameter measurements, p-values calculated by Mann-Whitney U-test, Statistically significant after Bonferroni correction for multiple comparisons (threshold is p = 0.0002), Abbreviations: SBP: Systolic blood pressure, DBP: diastolic blood pressure, LVEF: Left ventricular ejection fraction, LVEDd: Left ventricular end-diastolic diameter, IVSd: Interventricular septum thickness , PWED: Posterior wall thickness, LVMI: Left ventricular mass index, LAVES (4CH): Left atrial volume four-chambers, LAVES (2CH): Left atrial volume two-chambers, LAVES mean: Left atrial volume mean from LAVES (4CH) and LAVES (2CH), LAVI: Left atrial volume index, TAPSE: Tricuspid annular plane systolic excursion, MAPSE: Mitral annular plane systolic excursion

**Supplemental Table 6.** Investigation of differences in blood pressure, heart rate, electrocardiographic and echocardiographic parameters between participants with an EDs consumption of ≥6 mg caffeine from EDs/ kg BW/ day (n = 26) compared to the control group (n = 160)

|  |  | **Chronic high ED consumers** |  | **Control group (n=160)** |  |
| --- | --- | --- | --- | --- | --- |
|  |  | ≥ 6.00 mg caffeine from EDs /kg BW/day (n=26) |  | No ED consumption |  |
|  | n | Median (Q1-Q3) | n | Median (Q1-Q3) | *p-Values* |
| SBP [mmHg] | 26 | 120.0 (113.5-125.0) | 160 | 118.3 (110.3-128.3) | *0.92* |
| DBP [mmHg] | 26 | 77.5 (68.0-82.0) | 160 | 74.0 (68.3-80.5) | *0.60* |
| Heart rate [BPM] | 26 | 73.0 (65.0-77.0) | 160 | 73.0 (64.0-84.0) | *0.95* |
| **Electrocardiographic parameters** |  |  |  |  |  |
| PQ interval [ms] | 26 | 139.0 (130.0-150.0) | 159 | 140.0 (130.0-150.0) | *0.68* |
| QTc interval [ms] | 26 | 368.0 (346.0-378.0) | 159 | 364.0 (344.0-384.0) | *0.65* |
| QRS duration [ms] | 26 | 91.0 (86.0-96.0) | 159 | 88.0 (82.0-94.0) | *0.89* |
| **Echocardiographic parameters** |  |  |  |  |  |
| LVEF [%] | 26 | 60.5 (56.0-63.0) | 160 | 61.0 (58.0-64.0) | *0.91* |
| LVEDd [mm] | 26 | 44.0 (42.0-48.0) | 159 | 44.0 (41.0-48.0) | *0.86* |
| IVSd [mm] | 26 | 9.00 (7.90-10.0) | 159 | 8.00 (7.00-9.00) | *0.60* |
| PWED [mm] | 26 | 7.00 (7.00-8.00) | 159 | 8.00 (7.00-9.00) | *0.22* |
| LVMI [g/m^2^] | 26 | 72.7 (60.3-82.1) | 159 | 68.3 (56.4-80.3) | *0.55* |
| LAVES (4CH) [ml] | 26 | 29.7 (25.0-37.0) | 155 | 31.6 (25.0-40.8) | *0.45* |
| LAVES (2CH) [ml] | 26 | 36.6 (28.0-47.0) | 155 | 36.0 (28.0-46.4) | *0.26* |
| LAVES mean [ml] | 26 | 33.3 (27.3-42.0) | 155 | 34.5 (28.0-43.0) | *0.32* |
| LAVI [ml/ml^2^] | 26 | 21.0 (17.1-26.1) | 154 | 21.0 (18.0-26.0) | *0.15* |
| TAPSE [mm] | 26 | 23.0 (20.0-27.0) | 158 | 23.0 (21.0-25.0) | *0.12* |
| MAPSE [mm] | 26 | 16.0 (14.0-17.0) | 158 | 16.0 (14.0-17.0) | *0.16* |
| E-Wave [cm/s] | 26 | 84.0 (81.0-92.0) | 158 | 90.0 (80.0-99.0) | *0.07* |
| A-Wave [cm/s] | 26 | 50.5 (42.0-61.0) | 158 | 50.0 (41.0-58.0) | *0.13* |
| E / A | 25 | 1.61 (1.46-1.93) | 158 | 1.80 (1.50-2.20) | *0.88* |
| e‘ lateral [cm/s] | 25 | 17.0 (15.0-20.4) | 155 | 18.0 (16.0-20.4) | *0.73* |
| e‘ septal [cm/s] | 25 | 14.0 (13.0-15.0) | 155 | 14.0 (13.0-16.0) | *0.95* |
| e‘ mean [cm/s] | 25 | 15.5 (14.0-18.0) | 154 | 16.0 (14.5-18.0) | *0.76* |
| E / e’ lateral | 25 | 5.06 (3.92-5.63) | 153 | 4.83 (4.29-5.91) | *0.20* |
| E / e’ septal | 25 | 5.94 (5.54-7.00) | 153 | 6.20 (5.40-7.27) | *0.08* |
| E / e’ mean | 25 | 5.52 (4.65-6.07) | 152 | 5.51 (4.87-6.36) | *0.14* |
| Global longitudinal strain [%] | 25 | -20.0 (-21.3 to -18.1) | 139 | -20.0 (-22.0 to -18.0) | *0.41* |

Varying numbers of study participants in the cardiological parameters are caused by missing parameter measurements, p-values calculated by Mann-Whitney U-test, Statistically significant after Bonferroni correction for multiple comparisons (threshold is p = 0.0002), Abbreviations: SBP: Systolic blood pressure, DBP: diastolic blood pressure, LVEF: Left ventricular ejection fraction, LVEDd: Left ventricular end-diastolic diameter, IVSd: Interventricular septum thickness , PWED: Posterior wall thickness, LVMI: Left ventricular mass index, LAVES (4CH): Left atrial volume four-chambers, LAVES (2CH): Left atrial volume two-chambers, LAVES mean: Left atrial volume mean from LAVES (4CH) and LAVES (2CH), LAVI: Left atrial volume index, TAPSE: Tricuspid annular plane systolic excursion, MAPSE: Mitral annular plane systolic excursion

**Supplemental Table 7.** Investigation of differences in blood pressure, heart rate, electrocardiographic and echocardiographic parameters between participants with an EDs consumption of ≥ 2 years (n = 83) compared to the control group (n = 160)

|  |  | **Chronic high ED consumers** |  | **Control group (n=160)** |  |
| --- | --- | --- | --- | --- | --- |
|  |  | More than 2 years consumption (n=83) |  | No ED consumption |  |
|  | n | Median (Q1-Q3) | n | Median (Q1-Q3) | *p-Values* |
| SBP [mmHg] | 83 | 120.5 (113.5-128.5) | 160 | 118.3 (110.3-128.3) | *0.16* |
| DBP [mmHg] | 83 | 76.0 (68.5-82.5) | 160 | 74.0 (68.3-80.5) | *0.32* |
| Heart rate [BPM] | 83 | 75.0 (69.0-85.0) | 160 | 73.0 (64.0-84.0) | *0.19* |
| **Electrocardiographic parameters** |  |  |  |  |  |
| PQ interval [ms] | 83 | 140.0 (128.0-150.0) | 159 | 140.0 (130.0-150.0) | *0.59* |
| QTc interval [ms] | 83 | 358.0 (342.0-380.0) | 159 | 364.0 (344.0-384.0) | *0.27* |
| QRS duration [ms] | 83 | 88.0 (82.0-84.0) | 159 | 88.0 (82.0-94.0) | *0.77* |
| **Echocardiographic parameters** |  |  |  |  |  |
| LVEF [%] | 82 | 60.0 (57.0-64.0) | 160 | 61.0 (58.0-64.0) | *0.33* |
| LVEDd [mm] | 82 | 44.0 (41.9-48.0) | 159 | 44.0 (41.0-48.0) | *0.47* |
| IVSd [mm] | 82 | 9.00 (7.80-10.0) | 159 | 8.00 (7.00-9.00) | *0.10* |
| PWED [mm] | 82 | 8.00 (7.00-9.00) | 159 | 8.00 (7.00-9.00) | *0.61* |
| LVMI [g/m^2^] | 82 | 68.6 (56.6-81.6) | 159 | 68.3 (56.4-80.3) | *0.55* |
| LAVES (4CH) [ml] | 82 | 32.8 (25.2-41.5) | 155 | 31.6 (25.0-40.8) | *0.67* |
| LAVES (2CH) [ml] | 80 | 38.9 (28.0-45.5) | 155 | 36.0 (28.0-46.4) | *0.29* |
| LAVES mean [ml] | 80 | 36.9 (28.0-45.5) | 155 | 34.5 (28.0-43.0) | *0.47* |
| LAVI [ml/ml^2^] | 80 | 21.3 (17.0-24.8) | 154 | 21.0 (18.0-26.0) | *0.70* |
| TAPSE [mm] | 82 | 23.0 (20.0-25.0) | 158 | 23.0 (21.0-25.0) | *0.84* |
| MAPSE [mm] | 82 | 16.0 (14.0-17.2) | 158 | 16.0 (14.0-17.0) | *0.88* |
| E-Wave [cm/s] | 81 | 84.0 (76.0-95.0) | 158 | 90.0 (80.0-99.0) | *0.01* |
| A-Wave [cm/s] | 81 | 50.0 (42.0-59.0) | 158 | 50.0 (41.0-58.0) | *0.58* |
| E / A | 81 | 1.62 (1.44-1.95) | 158 | 1.80 (1.50-2.20) | *0.03* |
| e‘ lateral [cm/s] | 78 | 18.0 (16.0-20.0) | 155 | 18.0 (16.0-20.4) | *0.91* |
| e‘ septal [cm/s] | 79 | 14.0 (13.0-16.0) | 155 | 14.0 (13.0-16.0) | *0.09* |
| e‘ mean [cm/s] | 78 | 16.0 (15.0-18.0) | 154 | 16.0 (14.5-18.0) | *0.52* |
| E / e’ lateral | 77 | 4.66 (4.12-5.33) | 153 | 4.83 (4.29-5.91) | *0.06* |
| E / e’ septal | 78 | 6.00 (5.29-6.91) | 153 | 6.20 (5.40-7.27) | *0.30* |
| E / e’ mean | 77 | 5.36 (4.65-6.00) | 152 | 5.51 (4.87-6.36) | *0.10* |
| Global longitudinal strain [%] | 76 | -20.0 (-21.4 to -18.0) | 139 | -20.0 (-22.0 to -18.0) | *0.56* |

Varying numbers of study participants in the cardiological parameters are caused by missing parameter measurements, p-values calculated by Mann-Whitney U-test, Statistically significant after Bonferroni correction for multiple comparisons (threshold is p = 0.0002), Abbreviations: SBP: Systolic blood pressure, DBP: diastolic blood pressure, LVEF: Left ventricular ejection fraction, LVEDd: Left ventricular end-diastolic diameter, IVSd: Interventricular septum thickness , PWED: Posterior wall thickness, LVMI: Left ventricular mass index, LAVES (4CH): Left atrial volume four-chambers, LAVES (2CH): Left atrial volume two-chambers, LAVES mean: Left atrial volume mean from LAVES (4CH) and LAVES (2CH), LAVI: Left atrial volume index, TAPSE: Tricuspid annular plane systolic excursion, MAPSE: Mitral annular plane systolic excursion

**Supplemental Table 8.** Stratified analyses (median-split) investigating differences in blood pressure, heart rate, electrocardiographic and echocardiographic parameters between participants with total intake of caffeine from EDs and other caffeinated beverages ≤ 6.65 mg caffeine /kg BW/day or > 6.65 mg caffeine /kg BW/day compared to the control group

|  |  | **Chronic high ED consumers** | | |  | **Control group (n=160)** |  |  |
| --- | --- | --- | --- | --- | --- | --- | --- | --- |
|  |  | ≤ 6.65 mg caffeine /kg BW/day (n=48) |  | > 6.65 mg caffeine /kg BW/day (n=49) |  | No ED consumption |  |  |
|  | n | Median (Q1-Q3) | n | Median (Q1-Q3) | n | Median (Q1-Q3) | *p-Values ^a^* | *p-Values ^b^* |
| Total caffeine intake  [mg caffeine/ kg BW/ day] | 48 | 5.24 (4.37-6.09) | 49 | 8.63 (7.62-10.4) | 160 | 0.05 (0.02-0.11) |  |  |
| Intake caffeine from EDs  [mg caffeine from EDs/ kg BW/ day] | 48 | 3.78 (3.30-4.55) | 49 | 6.08 (4.50-7.52) | - | - |  |  |
| Intake of caffeine from caffeinated beverages (not EDs)  [mg caffeine/ kg BW/ day] | 48 | 1.16 (0.60-1.63) | 49 | 2.44 (1.33-3.99) | 160 | 0.05 (0.02-0.11) |  |  |
| SBP [mmHg] | 48 | 119.0 (111.0- 128.0) | 49 | 122.0 (116.0-129.0) | 160 | 118.3 (110.3-128.3) | *0.47* | *0.10* |
| DBP [mmHg] | 48 | 77.8 (69.0-84.0) | 49 | 73.5 (68.5-81.5) | 160 | 74.0 (68.3-80.5) | *0.12* | *0.62* |
| Heart rate [BPM] | 48 | 75.0 (70.5-87.5) | 49 | 74.0 (66.0-82.0) | 160 | 73.0 (64.5-84.0) | *0.18* | *0.76* |
| **Electrocardiographic parameters** | | |  |  |  |  |  |  |
| PQ interval [ms] | 48 | 140.0 (129.0-149.0) | 49 | 140.0 (130.0-150.0) | 158 | 140.0 (130.0-150.0) | *0.67* | *0.61* |
| QTc interval [ms] | 48 | 357.0 (344.0-379.0) | 49 | 364.0 (340.0-382.0) | 159 | 364.0 (344.0-384.0) | *0.32* | *0.52* |
| QRS duration [ms] | 48 | 84.0 (80.0-92.0) | 49 | 90.0 (82.0-96.0) | 159 | 88.0 (82.0-94.0) | *0.14* | *0.22* |
| **Echocardiographic parameters** | | |  |  |  |  |  |  |
| LVEF [%] | 47 | 60.0 (56.0-64.0) | 49 | 60.0 (58.0-64.0) | 160 | 61.0 (58.0-64.0) | *0.41* | *0.78* |
| LVEDd [mm] | 47 | 44.0 (41.0-48.0) | 49 | 44.0 (42.0-48.5) | 159 | 44.0 (41.0-48.0) | *0.72* | *0.38* |
| IVSd [mm] | 47 | 9.00 (7.00-10.0) | 49 | 9.00 (8.00-10.0) | 159 | 8.00 (7.00-9.00) | *0.11* | *0.19* |
| PWED [mm] | 47 | 7.00 (6.10-9.00) | 49 | 8.00 (7.00-8.60) | 159 | 8.00 (7.00-9.00) | *0.30* | *0.77* |
| LVMI [g/m^2^] | 47 | 68.1 (56.0-81.6) | 49 | 71.5 (59.0-82.0) | 159 | 68.3 (56.4-80.3) | *0.82* | *0.34* |
| LAVES (4CH) [ml] | 47 | 32.0 (24.5-40.0) | 48 | 32.8 (26.0-41.4) | 155 | 31.6 (25.0-40.8) | *0.98* | *0.65* |
| LAVES (2CH) [ml] | 45 | 38.0 (27.0-46.8) | 48 | 38.1 (29.5-50.0) | 155 | 36.0 (28.0-46.4) | *0.62* | *0.31* |
| LAVES mean [ml] | 45 | 35.5 (27.5-43.1) | 48 | 36.4 (28.3-45.3) | 155 | 34.5 (28.0-43.0) | *0.87* | *0.50* |
| LAVI [ml/ml^2^] | 45 | 20.9 (17.0-23.7) | 48 | 22.6 (17.1-28.8) | 154 | 21.0 (18.0-25.0) | *0.57* | *0.74* |
| TAPSE [mm] | 47 | 22.0 (20.0-25.0) | 49 | 23.0 (21.0-26.0) | 158 | 23.0 (21.0-25.0) | *0.18* | *0.14* |
| MAPSE [mm] | 47 | 15.3 (14.0-17.8) | 49 | 16.0 (15.0-17.2) | 158 | 16.0 (14.0-17.0) | *0.82* | *0.29* |
| E-Wave [cm/s] | 47 | 86.0 (74.0-95.0) | 48 | 85.0 (80.0-97.0) | 158 | 90.0 (80.0-99.0) | *0.08* | *0.27* |
| A-Wave [cm/s] | 47 | 51.0 (43.0-59.0) | 48 | 49.5 (41.5-60.0) | 158 | 50.0 (41.0-58.0) | *0.54* | *0.89* |
| E / A | 47 | 1.65 (1.45-1.95) | 48 | 1.63 (1.46-2.13) | 158 | 1.80 (1.50-2.20) | *0.14* | *0.32* |
| e‘ lateral [cm/s] | 46 | 18.5 (16.0-20.0) | 45 | 17.0 (15.0-20.4) | 155 | 18.0 (16.0-20.4) | *0.93* | *0.50* |
| e‘ septal [cm/s] | 47 | 14.0 (12.0-16.0) | 45 | 14.0 (13.0-15.0) | 155 | 14.0 (13.0-16.0) | *0.10* | *0.13* |
| e‘ mean [cm/s] | 46 | 16.0 (15.0-17.5) | 45 | 15.5 (14.5-18.0) | 154 | 16.0 (14.5-18.0) | *0.47* | *0.25* |
| E / e’ lateral | 46 | 4.87 (4.12-5.50) | 44 | 4.85 (4.41-5.54) | 152 | 4.83 (4.29-5.89) | *0.35* | *0.63* |
| E / e’ septal | 47 | 6.07 (5.38-7.14) | 44 | 6.16 (5.43-6.96) | 153 | 6.20 (5.40-7.27) | *0.76* | *0.93* |
| E / e’ mean | 46 | 5.50 (4.65-6.07) | 44 | 5.44 (4.79-6.04) | 152 | 5.51 (4.87-6.36) | *0.55* | *0.65* |
| Global longitudinal strain [%] | 39 | -19.0 (-21.2 to -17.0) | 46 | -20.5 (-21.6 to -18.1) | 139 | -20.0 (-22.0 to -18.0) | *0.24* | *0.63* |

Varying numbers of study participants in the cardiological parameters are caused by missing parameter measurements, ^a^ Comparison between chronic high consumers with ≤ 6.65 mg caffeine/ kg BW/ day in comparison to control group, ^b^ Comparison between chronic high consumers with > 6.65 mg caffeine/ kg BW/ day in comparison to control group, P-values calculated by Mann-Whitney U-test, Statistically significant after Bonferroni correction for multiple comparisons (threshold is p = 0.0002), Other caffeinated beverages: coffee drinks, black/green tea, drinking chocolate, cola/ mate, iced tea or pre-workout boosters, Abbreviations: SBP: Systolic blood pressure, DBP: diastolic blood pressure, LVEF: Left ventricular ejection fraction, LVEDd: Left ventricular end-diastolic diameter, IVSd: Interventricular septum thickness , PWED: Posterior wall thickness, LVMI: Left ventricular mass index, LAVES (4CH): Left atrial volume four-chambers, LAVES (2CH): Left atrial volume two-chambers, LAVES mean: Left atrial volume mean from LAVES (4CH) and LAVES (2CH), LAVI: Left atrial volume index, TAPSE: Tricuspid annular plane systolic excursion, MAPSE: Mitral annular plane systolic excursion

**Supplemental Table 9.** Investigation of selection bias between all invited participants to study phase 2 with cardiological examination compared to all invited participants without cardiological examination stratified by chronic high ED consumption or control group

|  | **Chronic high ED consumption** | | | | **Control group** | | | |
| --- | --- | --- | --- | --- | --- | --- | --- | --- |
|  | n | Invited  with cardiological examination (n=97) | Invited  without cardiological examination  (n=187) | *p-Values* | n | Invited  with  cardiological examination (n=160) | Invited  without cardiological examination (n=264) | *p-Values* |
| **Gender** | 284 |  |  | *0.20* | 424 |  |  | *0.003* |
| Male |  | 46.4% (45) | 55.1% (103) |  |  | 48.8% (78) | 62.5% (165) |  |
| Female |  | 48.5% (47) | 42.8% (80) |  |  | 49.4% (79) | 37.5% (99) |  |
| Divers |  | 5.1% (5) | 2.1% (4) |  |  | 1.9% (3) | 0.0% (0) |  |
| Age [years] | 284 | 16.0 (16.0-17.0) | 16.0 (16.0-17.0) | *0.45* | 424 | 16.0 (15.0-16.5) | 16.0 (15.0-17.0) | *0.23* |
| BMI [kg/m^2^] | 284 | 21.7 (19.7-24.6) | 21.4 (19.3-24.2) | *0.35* | 424 | 20.2 (18.6-22.7) | 20.6 (19.0-23.3) | *0.07* |
| **Type of school** | 284 |  |  | *0.31* |  |  |  | *0.0005* |
| Grammar school |  | 14.3% (14) | 13.4% (25) |  |  | 33.8% (54) | 33.3% (88) |  |
| Secondary school |  | 55.7% (54) | 47.6% (89) |  |  | 55.6% (89) | 41.3% (109) |  |
| Vocational high school center |  | 29.9% (29) | 39.0% (73) |  |  | 10.6% (17) | 25.4% (67) |  |
| Born in Germany | 281 | 82.3% (79) | 89.7% (166) | *0.08* | 423 | 91.9% (147) | 85.6% (225) | *0.05* |
| **Physical activity** | 280 |  |  | *0.24* | 415 |  |  | *0.35* |
| Never |  | 13.7% (13) | 13.0% (24) |  |  | 8.9% (14) | 11.7% (30) |  |
| <1x per week |  | 14.7% (14) | 13.0% (24) |  |  | 12.0% (19) | 8.2% (21) |  |
| 1x per week |  | 12.6% (12) | 24.3% (45) |  |  | 20.9% (33) | 16.0% (41) |  |
| 2-6x per week |  | 48.4% (46) | 40.0% (74) |  |  | 47.5% (75) | 54.1% (139) |  |
| daily |  | 10.5% (10) | 9.7% (18) |  |  | 10.8% (17) | 10.1% (26) |  |
| **Smoking** | 274 |  |  | *0.02* | 419 |  |  | *0.72* |
| Smoking |  | 50.6% (46) | 58.5% (107) |  |  | 1.9% (3) | 2.3% (6) |  |
| Ex-Smoking |  | 14.3% (13) | 21.3% (39) |  |  | 3.1% (5) | 4.6% (12) |  |
| Non-Smoking |  | 35.2% (32) | 20.2% (37) |  |  | 95.0% (151) | 93.1% (242) |  |
| Ever smoked Weed [yes] | 274 | 44.2% (42) | 54.2 % (97) | *0.12* | 421 | 5.0 % (8) | 5.8 % (15) | *0.74* |
| **Alcohol consumption** |  |  |  |  |  |  |  |  |
| Ever consumed alcohol [yes] | 280 | 75.0% (72) | 82.1% (151) | *0.16* | 417 | 46.2% (73) | 39.8 (103) | *0.20* |
| Alcohol consumption last 12 month | 219 |  |  | *0.83* | 148 |  |  | *0.04* |
| 1x per month or less |  | 25.4% (18) | 32.4% (48) |  |  | 76.6% (49) | 76.2% (64) |  |
| 2–3x per month |  | 29.6% (21) | 23.0% (34) |  |  | 15.6% (10) | 3.6% (3) |  |
| 1x per week |  | 11.3% (8) | 9.5% (14) |  |  | 7.8% (5) | 11.9% (10) |  |
| 2x per week |  | 15.4% (11) | 15.5% (23) |  |  | - | 4.8% (4) |  |
| 3x per week |  | 11.3% (8) | 9.5% (14) |  |  | - | 1.2% (1) |  |
| 4x per week |  | 4.2% (3) | 4.1% (6) |  |  | - | - |  |
| 5–6x per week |  | 2.8% (2) | 4.1% (6) |  |  | - | - |  |
| daily |  | - | 2.0% (3) |  |  | - | 2.4% (2) |  |
| **Sleep duration** Weekdays | 282 |  |  | *0.47* | 421 |  |  | *0.23* |
| < 5 h |  | 17.5% (17) | 22.2% (41) |  |  | 1.9% (3) | 4.9% (13) |  |
| 5-6 h |  | 36.1% (35) | 29.2% (54) |  |  | 10.8% (17) | 16.0% (42) |  |
| 6-7 h |  | 27.8% (27) | 22.7% (42) |  |  | 28.5% (45) | 21.3% (56) |  |
| 7-8 h |  | 13.4% (13) | 17.3% (32) |  |  | 39.2% (62) | 40.3% (106) |  |
| 8-9 h |  | 3.1% (3) | 7.0% (13) |  |  | 17.1% (27) | 12.9% (34) |  |
| 9-10 h |  | 2.1% (2) | 1.1% (2) |  |  | 2.5% (4) | 3.8% (10) |  |
| 10-11 h |  | - | 0.5% (1) |  |  | - | 0.4% (1) |  |
| >11 h |  | - | - |  |  | - | 0.4% (1) |  |
| **Sleep duration** Weekends | 283 |  |  | *0.38* | 421 |  |  | *0.72* |
| < 5 h |  | 5.2% (5) | 10.2% (19) |  |  | 0% (0) | 1.5% (4) |  |
| 5-6 h |  | 7.2% (7) | 9.7% (18) |  |  | 1.9% (3) | 2.3% (6) |  |
| 6-7 h |  | 10.3% (10) | 8.1% (15) |  |  | 5.7% (9) | 6.1% (16) |  |
| 7-8 h |  | 10.3% (10) | 14.0% (26) |  |  | 15.2% (24) | 12.2% (32) |  |
| 8-9 h |  | 23.7% (23) | 16.1% (30) |  |  | 32.9% (52) | 33.4% (88) |  |
| 9-10 h |  | 20.6% (20) | 18.3% (34) |  |  | 27.2% (43) | 28.1% (74) |  |
| 10-11 h |  | 14.4% (14) | 18.8% (35) |  |  | 13.3% (21) | 10.7% (28) |  |
| >11 h |  | 8.3% (8) | 4.8% (9) |  |  | 3.8% (6) | 5.7% (75) |  |

Variables expressed as % (n) or median (IQR), P-values calculated by Mann-Whitney U-test or chi-square test
